# An Anomalous Type IV Secretion System in *Rickettsia* Is Evolutionarily Conserved

**DOI:** 10.1371/journal.pone.0004833

**Published:** 2009-03-12

**Authors:** Joseph J. Gillespie, Nicole C. Ammerman, Sheila M. Dreher-Lesnick, M. Sayeedur Rahman, Micah J. Worley, Joao C. Setubal, Bruno S. Sobral, Abdu F. Azad

**Affiliations:** 1 Virginia Bioinformatics Institute at Virginia Tech, Blacksburg, Virginia, United States of America; 2 Department of Microbiology and Immunology, University of Maryland School of Medicine, Baltimore, Maryland, United States of America; 3 Department of Biology, University of Louisville, Louisville, Kentucky, United States of America; 4 Department of Computer Science, Virginia Tech, Blacksburg, Virginia, United States of America; Charité-Universitätsmedizin Berlin, Germany

## Abstract

**Background:**

Bacterial type IV secretion systems (T4SSs) comprise a diverse transporter family functioning in conjugation, competence, and effector molecule (DNA and/or protein) translocation. Thirteen genome sequences from *Rickettsia*, obligate intracellular symbionts/pathogens of a wide range of eukaryotes, have revealed a reduced T4SS relative to the *Agrobacterium tumefaciens* archetype (*vir*). However, the *Rickettsia* T4SS has not been functionally characterized for its role in symbiosis/virulence, and none of its substrates are known.

**Results:**

Superimposition of T4SS structural/functional information over previously identified *Rickettsia* components implicate a functional *Rickettsia* T4SS. *virB4*, *virB8* and *virB9* are duplicated, yet only one copy of each has the conserved features of similar genes in other T4SSs. An extraordinarily duplicated VirB6 gene encodes five hydrophobic proteins conserved only in a short region known to be involved in DNA transfer in *A. tumefaciens*. *virB1*, *virB2* and *virB7* are newly identified, revealing a *Rickettsia* T4SS lacking only *virB5* relative to the *vir* archetype. Phylogeny estimation suggests vertical inheritance of all components, despite gene rearrangements into an archipelago of five islets. Similarities of *Rickettsia* VirB7/VirB9 to ComB7/ComB9 proteins of ε-proteobacteria, as well as phylogenetic affinities to the *Legionella lvh* T4SS, imply the Rickettsiales ancestor acquired a *vir*-like locus from distantly related bacteria, perhaps while residing in a protozoan host. Modern modifications of these systems likely reflect diversification with various eukaryotic host cells.

**Conclusion:**

We present the *rvh* (Rickettsiales *vir* homolog) T4SS, an evolutionary conserved transporter with an unknown role in rickettsial biology. This work lays the foundation for future laboratory characterization of this system, and also identifies the *Legionella lvh* T4SS as a suitable genetic model.

## Introduction

**Figure 1:**
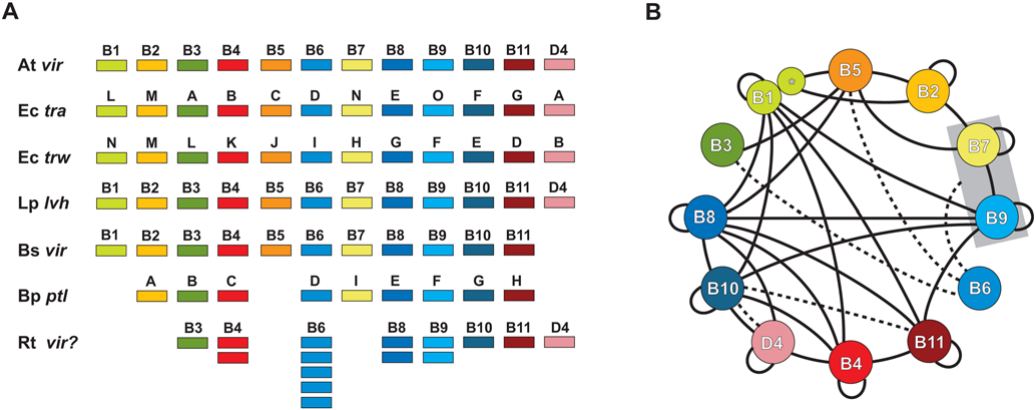
Characteristics of the archetypal bacterial type IV secretion system (type A). (A) Schema depicting several of the type A T4SSs used in this investigation for comparison with the *Rickettsia* T4SS. At *vir* = *Agrobacterium tumefaciens* Ti plasmid; Ec *tra* = *Escherichia coli* IncN plasmid pKM101; Ec *trw* = *Escherichia coli* plasmid R388; Lp *lvh* = *Legionella pneumophila*; Bs *vir* = *Brucella suis*; Bp *ptl* = *Bordetella pertussis*; Rt *vir*? = *Rickettsia typhi*. (B) Map of Vir-Vir *physical* interactions based on a survey of the literature. Specific studies demonstrating the mapped interactions are discussed in the text and in supporting Document S1. Solid black lines depict direct physical interactions. Dashed lines depict energetic effects of ATP hydrolysis of VirD4 and VirB11 on VirB10 [100], as well as the stabilization effect of VirB6 on VirB3, VirB5, and VirB7 dimerization [184] and the VirB7/VirB9 heterodimer [131]. VirB1 and VirB1* are modeled accordingly [65] and the VirB7/VirB9 complex is boxed to illustrate interactions with the heterodimer rather than individual components.

Type IV secretion systems (T4SSs) are multi-component membrane-spanning transporters present in many Gram-negative bacteria, including medically and agriculturally important species. Derived from ancient conjugation machineries [1], T4SSs are presumably used by many species for the exchange of genetic material [2], [3], [4], [5]. However, T4SSs have garnered significant attention for their role in pathogenesis, acting as syringes that inject virulence factors into eukaryotic host cells [6]. These translocated virulence factors, more commonly referred to as ‘effector molecules’ (DNA, protein or nucleoprotein complexes), have a broad range of host-altering functions, such as highjacking of vesicular trafficking [7], cytoskeletal modification [8], ubiquitination system exploitation [9], and genome introgression [10], [11], [12], [13], [14], [15]. T4SSs are found in a variety of etiological agents of human disease, including *Bordetella pertussis* (whooping cough), *Coxiella burnetii* (Q fever), *Brucella* spp. (brucellosis), *Bartonella henselae* (cat-scratch disease), *Campylobacter jejuni* (gastroenteritis), *Helicobacter pylori* (gasteric ulcers), and *Legionella pneumophila* (Legionnaires' disease) [16], [17], [18], [19], [20], [21], [22]. However, compilation of genome sequences has revealed the evolutionary persistence of T4SS genes (e.g., *vir*, *tra*, *trw*, etc.) from numerous Gram-negative (and now Gram-positive [23]) bacterial species, many of which have not been linked to pathogenesis. Consequently, the exact function(s) T4SSs bestow to the bacterial species that harbor them remain largely unknown, with roles in conjugation, pathogenesis, DNA uptake, or DNA release, or any combination of these processes, only determinable on an individual basis. Indeed, while considerable conservation of Vir and Vir-like components allows for comparison of T4SSs across divergent bacterial species [2], [6], [24], [25], [26], [27], [28], [29] (**Figure 1A**), identified protein effectors usually are conserved only in closely related species, suggesting common modes of type IV secretion have numerous divergent functions in both Gram-negative bacteria and Gram-positive bacteria, likely arising from multiple progenitors [26].

Much of what is known about the structure and function of T4SSs has been generated from paramount studies on the phytopathogen *Agrobacterium tumefaciens* [30]. Thus, the *virB/virD* T4SS of *A. tumefaciens* is considered the archetype to which most other *vir* and *vir*-like T4SSs (typically referred to as ‘type A’ T4SSs) are compared. In *A. tumefaciens*, the *virB* operon and *virD* locus on the Ti (tumor inducing) plasmid encode 12 single copy genes (*virB1_Ti_*-*virB11_Ti_*, *virD4_Ti_*) whose products comprise a molecular scaffold that spans the inner and outer membranes (IM and OM, respectively), culminating in a transfer pilus (T pilus) that protrudes from the bacteria [6], [31], [32], [33], [34], [35]. *A. tumefaciens* uses its *vir* T4SS, which is regulated by the VirA/VirG two-component system [36], to introduce a nucleoprotein complex, the T-strand DNA plus VirD2 and VirE2 proteins, into the nuclei of various host plant cells [37]. The byproduct of this interkingdom introgression is crown gall disease, which allows the bacteria to reside and multiply within a modified host niche [38].

The rudimentary functions of most components of the *A. tumefaciens vir* system have been characterized. VirD4_Ti_ is an IM ATPase that couples the nucleoprotein complex (and other substrates) to the T4SS channel [39], [40]. VirB4_Ti_ and VirB11_Ti_ are additional IM ATPases that presumably fuel the delivery of substrates to the core periplasmic-spanning channel [32], [41], [42], [43], [44], [45], [46], [47], [48], [49], [50], [51], [52]. VirB6_Ti_-VirB10_Ti_ form the core periplasmic-spanning channel [42], [45], [46], [51], [52], [53], [54], [55], [56], [57], [58], [59], [60], [61], while VirB2_Ti_ and VirB5_Ti_ are the major and minor constituents of the T pilus, respectively [42], [44], [46], [62], [63], [64]. VirB1_Ti_ is a member of the specialized lytic transglycosylase family of proteins implicated in the local degradation of peptidoglycan, allowing the channel to span the periplasm [42], [65], [66], [67]. The C-terminal portion of VirB1_Ti_ (VirB1*) is cleaved and functions in cell-cell contact and virulence [65]. Despite the vast body of research on *vir* systems, the function of VirB3_Ti_, an exported OM protein presumably involved in host cell attachment, remains unknown [42], [64]. Studies implicating the interaction of Vir components in *A. tumefaciens* as well as several other bacteria are rapidly growing and, coupled with recent structural studies, the architecture of the Vir scaffold is coming to light (**Figure 1B**).

A decade has passed since the first genome sequence was published for *Rickettsia*, obligate intracellular bacteria of the class *Alphaproteobacteria* [68], [69], [70]. The *R. prowazekii* str. Madrid E genome sequence (1.1 Mb) exposed an extraordinary trend toward genome reduction via pseudogenization, with major constituents of biosynthetic pathways deleted relative to other bacteria [71]. It also revealed a reduced T4SS as compared to the *virB/virD* T4SS of *A. tumefaciens*, with only six genes annotated as coding for Vir components (*virB4*, *virB8*-*virB11*, *virD4*). Interestingly, two of these Vir genes were duplicated (*virB4* and *virB9*, the latter gene annotated as *trbG*), and the arrangement of the Vir genes was non-canonical relative to most other T4SSs, being scattered in three well-separated locales of the genome. Subsequently sequenced rickettsial genomes have confirmed this atypical nature of the *Rickettisa* T4SS [72], [73], [74], [75], [76], [77], [78], [79], and consensus genome annotation revealed a T4SS devoid of only the VirB1, VirB2, VirB5, and VirB7 components [80], [81].

Despite intense laboratory effort, a paucity of characterized virulence factors exists for *Rickettsia* [82], with, to date, no genes identified as coding for effectors of the T4SS. This is surprising, as the virulent species of *Rickettsia* are of great interest both as emerging infectious diseases [83] and for their potential deployment as bioterrorism agents [84], [85]. Given the relatively close evolutionary relationship of *Agrobacterium* (Rhizobiales) and *Rickettsia* (Rickettsiales) within the *Alphaproteobacteria* tree [86], as well as the availability of 13 genome sequences of *Rickettsia* spp. (*R. bellii* str. RML369-C, *R. bellii* str. OSU 85 389, *R. canadensis* str. McKiel, *R. prowazekii* str. Madrid E, *R. typhi* str. Wilmington, *R. felis* str. URRWXCal2, *R. akari* str. Hartford, *R. massiliae* str. MTU5, *R. rickettsii* str. Sheila Smith CWPP, *R. rickettsii* str. Iowa, *R. conorii* str. Malish 7, *R. sibirica* str. 246, and *R. africae* str. ESF-5), it is timely to assess the atypical nature of the *Rickettsia* T4SS with an attempt to better understand the function (if any) this transporter has in rickettsial pathogenesis. We provide here a bioinformatic analysis of the *Rickettsia* T4SS, with each component evaluated in light of the latest structural and functional information from studies of *vir* and *vir*-like (e.g., *tra*, *trw*, *trb*, *ptl*) T4SSs from other bacteria. We address the nature of Vir gene duplication in *Rickettsia* and discuss the potential role the *vir* T4SS plays not only in rickettsial pathogenesis, but also lateral gene transfer (LGT). These results shed new light on the origin and function of the rickettsiae Vir-like genes and lay a much needed foundation for future laboratory assessment of the function of the *Rickettsia* T4SS.

## Results and Discussion

### Previously Identified *Rickettsia* T4SS Components

Fifteen highly conserved genes previously annotated in rickettsial genome sequencing projects have similar counterparts in the T4SSs of other bacteria (**Table 1**). These 15 genes comprise eight of the Vir components well characterized in the *A. tumefaciens* T4SS that are involved in substrate (DNA and/or protein) presentation (*virD4*), translocation energetics (*virB4*, *virB11*), mating channel structure (*virB6*, *virB8*-*virB10*), and host cell attachment (*virB3*) [26], [37]. Thus, consensus genome annotation supports prior findings that the *Rickettsia* T4SS is reduced in complexity relative to the *A. tumefaciens* model (missing genes encoding the components VirB1, VirB2, VirB5, and VirB7), with the various components present in three scattered genomic locales in all sequenced *Rickettsia* genomes (**Figure S1**). Accordingly, we evaluate the characteristics of each Vir component to propose that the T4SS in *Rickettsia* is a functional transporter. Important background information regarding these eight Vir components is compiled in **Document S1**.

**Table 1:** Relative Conservation of *Rickettsia* Vir Proteins to Vir and Vir-like Proteins from Other Bacteria.

Protein^1^	L query^2^	No. hits^3^	Distribution^4^					Other^5^
			α	β	γ	δ	ε	
VirD4	591	500	229	64	36	3	31	137 (6)
VirB4a	805	500	244	60	75	2	76	43 (15)
VirB4b	810	456	218	53	59	2	73	51 (28)
VirB11	334	500	200	109	107	18	8	58 (4)
VirB6a	1061	151	130	2	5	0	1	13 (2)
VirB6b	668	181	147	1	13	0	1	19
VirB6c	967	202	158	5	20	1	1	17 (2)
VirB6d	890	197	172	4	18	0	0	3 (1)
VirB6e	1154	247	174	14	35	1	9	14 (3)
VirB8a	228	101	65	4	11	1	3	17 (3)
VirB8b	242	153	80	10	36	0	16	11 (4)
VirB9a	247	411	203	74	73	3	36	22 (13)
VirB9b	158	342	197	53	63	1	8	20 (11)
VirB10	481	380	193	52	69	4	47	15 (11)
VirB3	95	80	65	1	7	0	6	7

1 Consensus annotation. Grouped by function: substrate presentation (VirD4), translocation energetics (VirB4, VirB11), mating channel (VirB6, VirB8-VirB10), attachment (VirB3).

2 Length (aa) of *R. typhi* query sequence.

3 Number of blastp subjects yielding significant alignments (maximum hits set to 500).

4 Distribution of subjects across five classes of proteobacteria.

5 Non-proteobacterial subjects, with number of plasmid encoded proteins in parentheses. Note: plasmid encoded Vir and Vir-like proteins were not assigned to a taxonomic class, hence some may be from plasmids found in proteobacteria.

#### Substrate presentation (VirD4)

The *Rickettsia* VirD4 gene is conserved across 13 genomes (8.1% div.), and the corresponding amino acid sequence (4.2% div.) is highly conserved when compared across divergent pathogenic bacterial type IV coupling proteins (T4CPs), particularly within the five conserved motifs of the two large C-terminal domains (CTDs) (**Figure S2**). Unpublished data from our laboratories also suggests that *Rickettsia* VirD4 is capable of binding putative effectors of the T4SS using a bacterial 2-hybrid assay.

#### Translocation energetics (VirB4 and VirB11)

**Figure 2:**
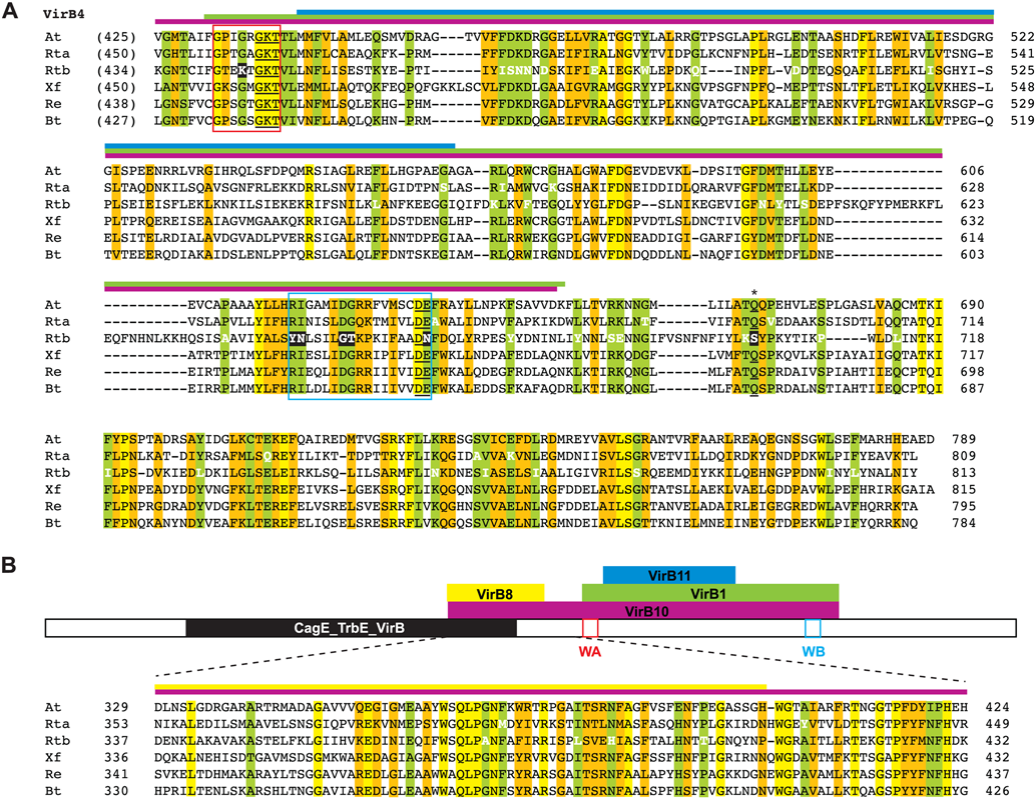
Characteristics and comparative analysis of VirB4 and VirB4-like amino acid sequences across six divergent bacterial species. Bars above alignment depict interactions between VirB4 and other Vir components as revealed by bacterial two-hybrid screen [208] with color scheme as follows: purple = VirB4-VirB10, yellow = VirB4-VirB8, green = VirB4-VirB1, blue = VirB4-VirB11. Residues colored white depict mutations in one rickettsial sequence in otherwise conserved positions in the alignment. Coordinates for each sequence are shown to the right in each block, with numbers in parentheses depicting flanking residues of the alignment not shown. Conservation color scheme as follows: yellow = identical; green = identical in five of six sequences; orange = identical in four of six sequences or a position comprised of only two residue states. See text for alignment details. Taxon abbreviations and associated NCBI accession numbers are as follows: At = *Agrobacterium tumefaciens* VirB4, NP_059802; Rta = *Rickettsia typhi* VirB4a, AAU03521; Rtb = *R. typhi* VirB4b, AAU04227; Xf = *Xylella fastidiosa* conjugal transfer protein (VirB4-like), Q9PHJ8; *Rhizobium etli* VirB4, Q8KIM8; *Bartonella tribocorum* VirB4, Q8GJ64. (A) Alignment of the C-terminal portion of VirB4 containing the conserved DNA-binding domain. Walker A (WA) and Walker B (WB) boxes are enclosed with red and blue boxes, respectively. The highly conserved Gln predicted to be involved in NTP-binding is denoted with an asterisk and underlined, with additional structurally proximal residues forming the purported NTP-binding cleft also underlined [209]. Seven residues within conserved NTP-binding domains mutated only in Rtb are shaded in black. (B) Schematic of the entire VirB4 protein depicting the interactions with four other Vir components [208]. Alignment depicts conservation outside of the conserved NTP binding domains. The CagE_TrbE_VirB domain (PF03135) plus WA and WB boxes are depicted in black, red and blue respectively.

Two VirB4 genes are present in all sequenced *Rickettsia* genomes (*virB4a* and *virB4b*), and both encoded proteins are large and similar in size and composition to VirB4 and VirB4-like proteins from other bacteria (**Figure 2**). In most *Rickettsia* genomes, *virB4a* is clustered with *virB3* and the *virB6* duplicates, while *virB4b* is not located near any other Vir genes (**Figure S1**). *virB4a* is more conserved across *Rickettsia* genomes than *virB4b* at the nucleotide (6.7% versus 9.2%) and amino acid (3.7% versus 9.9%) level, with more indels in VirB4b relative to VirB4a and other bacteria. In the protein alignment, 11 variable residues are VirB4a-specific, whereas 43 variable residues are unique to VirB4b, illustrating more conservation of VirB4a with non-*Rickettsia* proteins. Strikingly, seven of the VirB4b-specific substitutions alter critical residues in the NTP-binding cleft, which is essential for T-DNA export in *A. tumefaciens* [41], casting doubt on the ability of VirB4b to function as a T4SS ATPase.

The *Rickettsia* VirB11 gene is conserved across 13 genomes (9.7% div.) and the corresponding amino acid sequence (4.8% div.) is highly conserved when compared to VirB11 and VirB11-like proteins from other bacteria, particularly within the four conserved motifs of the CTD (**Figure S3**). Not surprisingly, the least conserved region in our alignment maps to the major difference between the two VirB11 crystals, a domain swap in the N-terminal domain (NTD)-CTD linker region of *Brucella suis* relative to *H. pylori* [87]. Like most other VirB11 and VirB11-like proteins, *Rickettsia* VirB4 contains the domain swap and likely forms an additional helix, αC2, in the linker B region that allows for additional interactions at the subunit-subunit interface. Finally, a search within all rickettsial genomes for the recently identified regulator of *H. pylori* VirB11, HP1451 [88], proved unsuccessful.

#### Mating channel structure (VirB6, VirB8-VirB10)

**Figure 3 pone-0004833-g003:**
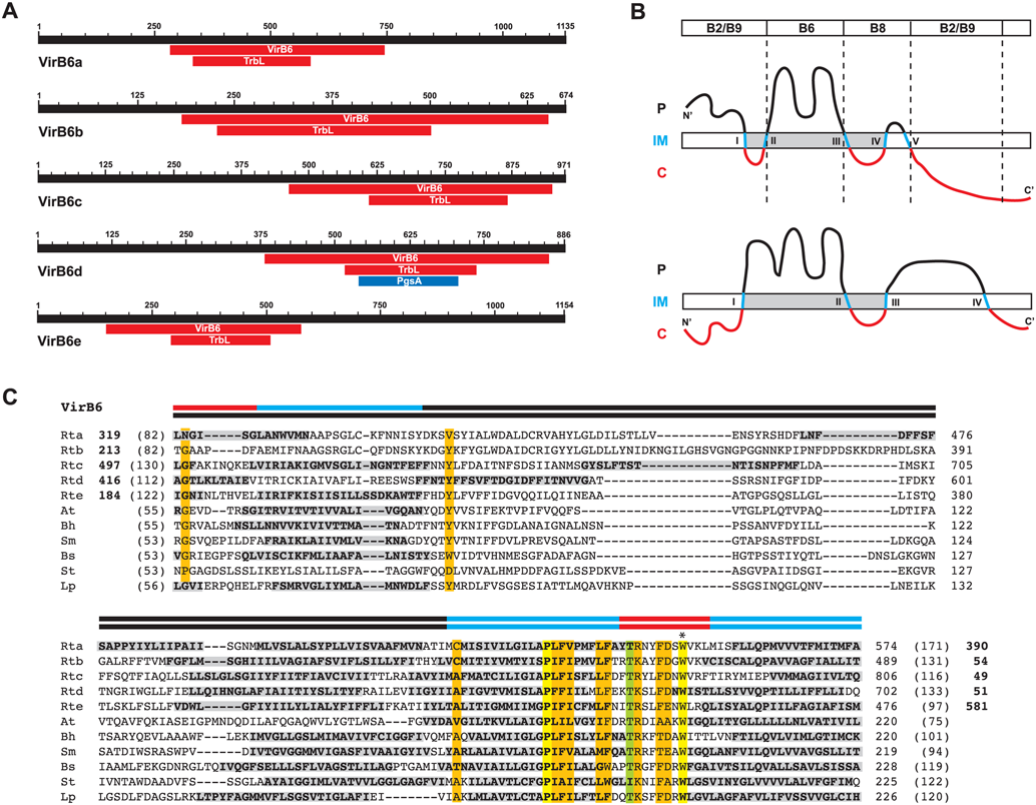
Characteristics of the highly duplicated VirB6-domain-containing proteins of *Rickettsia*, with comparison to VirB6 and VirB6-like amino acid sequences from six divergent bacterial species. (A) Schema of five *Rickettsia typhi* ORFs (Rta-e) containing putative VirB6 domains, as predicted by the Conserved Domains Database (NCBI) following blastp searches. All searches retrieved COG3704 (VirB6) and PF04610 (TrbL, TrbL/VirB6) domains, with Rtd also scoring limited similarity with COG0558 (PgsA, phosphatidylglycerophosphate synthase). (B) Competing topology models for *Agrobacterium tumefaciens* VirB6 in the IM (named here on the basis of the number of predicted TMS regions). Top: 5TMS model [89] with bar at top depicting regions of VirB6 required for formation of T-strand close contacts with several Vir components, as revealed by transfer DNA immunoprecipitation; bottom: 4TMS model [90]. Color scheme of protein as follows: black = periplasm, blue = IM, red = cytoplasm. TMS regions are labeled with Roman numerals sequentially from N- to C-terminus. Shaded (gray) portion depicts alignment in C. (C) Multiple sequence alignment of the central portion of the VirB6 domain of five (a–e) *R. typhi* ORFs and five VirB6 and VirB6-like sequences from diverse bacteria species. Bars above alignment depict shaded region in B, with competing models illustrated accordingly. Bold-shaded residues depict TMS regions as predicted by TMHMM [93]. The essential Trp residue [90], [92] is denoted with an asterisk above the alignment. Coordinates for each sequence are shown to the right in each block, with numbers in parentheses depicting flanking residues of the alignment not shown. Bold numbers for the rickettsial sequences depict regions not included in the VirB6 domain alignment. Conservation color scheme is the same as in Figure 2 legend, except orange = identical in seven of eleven sequences or a position comprised of only two residue states. See text for alignment details. Taxon abbreviations and associated NCBI accession numbers are as follows: Rta = *R. typhi* plasmid conjugal transfer protein VirB6 (VirB6a), YP_067002; Rtb = *R. typhi* TrbL/VirB6 plasmid conjugal transfer protein (VirB6b), YP_067001; Rtc = *R. typhi* plasmid conjugal transfer protein VirB6 (VirB6c), YP_067000; Rtd = *R. typhi* hypothetical protein (VirB6d), YP_066999; Rte = *R. typhi* plasmid conjugal transfer protein TrbL/VirB6 (VirB6e), YP_066998; At = *A. tumefaciens* VirB6, AAF77166; Bh = *Bartonella henselae* VirB6, AAF00944; Sm = *Sinorhizobium meliloti* VirB6, NP_435960; Bs = *Brucella suis* VirB6, NP_699271; St = *Salmonella typhimurium* (IncN plasmid R46) TraD, NP_511193; Lp = *Legionella pneumophila* LvhB6, AAM0824.

An extraordinary five copies of the VirB6 gene (*virB6a-e*) are found in most *Rickettsia* genomes (**Figure 3A**), with exception to *R. massiliae*, which is missing *virB6e* [72]. Additionally, *virB6d* is split in the *R. bellii* str. OSU 85 389 genome [81]. These findings, coupled with blast results (**Table 1**), suggest that these VirB6 genes are the most variable components of the *Rickettsia vir* system, particularly when the regions flanking the VirB6/TrbL domains are considered. Individual full length VirB6 genes are conserved across 13 genomes and range in nt divergence from 10.3% (12.4% aa) to 13.2% (15.2% aa); however, the five duplicates are extraordinarily different from one another, averaging ∼80% aa divergence in the VirB6/TrbL domains alone (alignment of full length proteins was confounded by variability in the flanking sequences). This lack of conservation, coupled with conflicting evidence and hypotheses for VirB6 channel structure formation [89], [90] (**Figure 3B**), makes it difficult to predict the functionality of any of the five duplicate VirB6-like proteins in *Rickettsia*. The regions flanking the VirB6/TrbL domains are particularly divergent across the five duplicates (**Figure 3A**) and are similar only in a few instances to the sister taxon of *Rickettsia*, *Orientia tsutsugamushi* (**Figure S4**). The contribution of additional flanking sequences to the structure and function of these expanded VirB6/TrbL domain-containing ORFs, especially regarding substrate transport to VirB2 and VirB9 in the OM, is worthy of exploration as they are seemingly unique to Rickettsiaceae (**Figure 3C**). Comparison of the VirB6/TrbL domains of all five VirB6 proteins to VirB6 and VirB6-like proteins from other bacteria supports previous observations that conservation within this domain is limited to the transmembrane-spanning (TMS)-cytoplasmic region involved in substrate transfer to VirB8 [89], [91]. Of the conserved residues in this region, the invariant Trp is essential [92] and required for polar localization of VirB6 [90]. Little conservation in sequence length and composition exists in the functionally important N-terminal large periplasmic region, and a TMS region prediction program [93] calculated at least one additional TMS region in this location of the domain in most analyzed taxa (**Figure 3C**).

**Figure 4 pone-0004833-g004:**
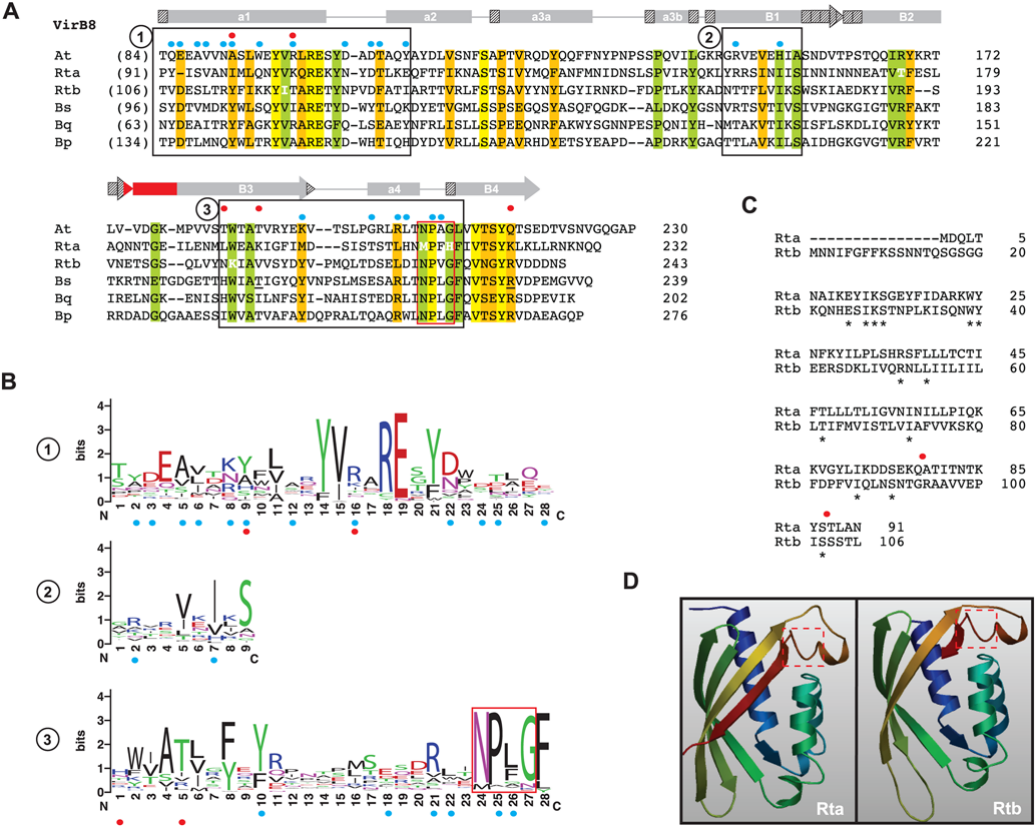
Comparative analysis of VirB8 and VirB8-like proteins with emphasis on two *Rickettsia* orthologs. (A) Multiple sequence alignment of the C-terminal regions of VirB8 and VirB8-like sequences corresponding to regions of the solved crystal structures of VirB8 from *Brucella suis* [95] and *Agrobacterium tumefaciens* [94]. Secondary structural model is shown above the alignment with arrows (β-strands B1–B4) and bars (α-helices A1–A4) with color scheme as follows: gray = agreement across both models, stippled = disagreement between models, red = alignment too variable to support either model. Structural notation follows the earlier model [95]. Red box encloses the highly conserved “NPxG” motif. Blue dots depict residues involved in VirB8 homo-dimerization [94], [95], [96], and red dots depict previously determined lethal mutants [96], [97]. In *B. suis* VirB8, residues implicated in interactions with VirB10 (T201) and VirB4 (R230) are underlined. Boxed regions are further described in B. Residues colored white depict mutations in one rickettsial sequence in otherwise conserved positions in the alignment. Coordinates for each sequence are shown to the right in each block, with numbers in parentheses depicting the number of flanking residues of the alignment not shown. Conservation color scheme is the same as in Figure 2 legend. See text for alignment details. Taxon abbreviations and associated NCBI accession numbers are as follows: At = *A. tumefaciens* VirB8, NP_059806; Rta = *Rickettsia typhi* VirB8a, YP_067240; Rtb = *R. typhi* VirB8b, YP_067242; Bs = *Brucella suis* VirB8, AAN33274; Bq = *Bartonella quintana* VirB8, AAM43802; Bp = *Bordetella pertussis* VirB8 homolog (PtlE), G47301. (B) Sequence logos [198], [199] illustrating consensus sequences for the three boxed regions in A generated across an alignment of 200 VirB and VirB-like bacterial sequences (sequences retrieved from NCBI with blastp using *R. typhi* VirB8a as a query). The “NPxG” motif is enclosed in red box. (C) Alignment of the N-terminal sequences of VirB8a and VirB8b from *R. typhi*. Identical residues are depicted with asterisks, with red dots as described above in A. (D) Predicted structure of Rta (left) and Rtb (right) using SWISS-MODEL v8.05 [200] with the *A. tumefaciens* VirB8 as a template (PDB ID code 2cc3A). The critical “NPxG” motif is enclosed in red dashed box.

Two VirB8 genes are present in *Rickettsia* genomes (*virB8a* and *virB8b*), separated by one small ORF (discussed below) and encoded on opposite strands (**Figure S1**). *virB8a* flanks *virB9a* on the minus strand while *virB8b* is within a larger *vir* cluster (*virB8b*-*virB9b*-*virB10*-*virB11*-*virD4*) on the plus strand. *virB8b* is more conserved across *Rickettsia* genomes than *virB8a* at the nucleotide (9% versus 10%) and amino acid (9.4% versus 13.4%) levels. Both VirB8 proteins are similar in size and composition to other VirB8 and VirB8-like proteins, with many residues within superimposed α-helices and β-strands of the solved crystal structures [94], [95] conserved in both proteins (**Figure 4A**). However, limited conservation is seen in residues implicated in dimerization [94], [95], [96] as well as positions previously demonstrated as lethal mutants [96], [97], even in an expanded comparison of 200 VirB8 and VirB8-like proteins (**Figure 4B**). Furthermore, VirB8a has two critical residues mutated within the highly conserved “NPxG” motif (XPxX across the 13 *Rickettsia* genomes), which is not expected to adopt its critical sharp turn confirmation between helix α4 and strand β4 if altered [95]. This, coupled with one of two essential residues mutated in the NTD (G78Q) relative to identical residues in VirB8b and VirB8_Ti_, as well as the extraordinary overall variation in the NTD (**Figure 4C**), casts doubt on VirB8a as a functional component of the *Rickettsia* T4SS. However, predicted structural models for both *Rickettsia* VirB8 proteins are similar to the VirB8_Ti_ crystal (**Figure 4D**).

**Figure 5 pone-0004833-g005:**
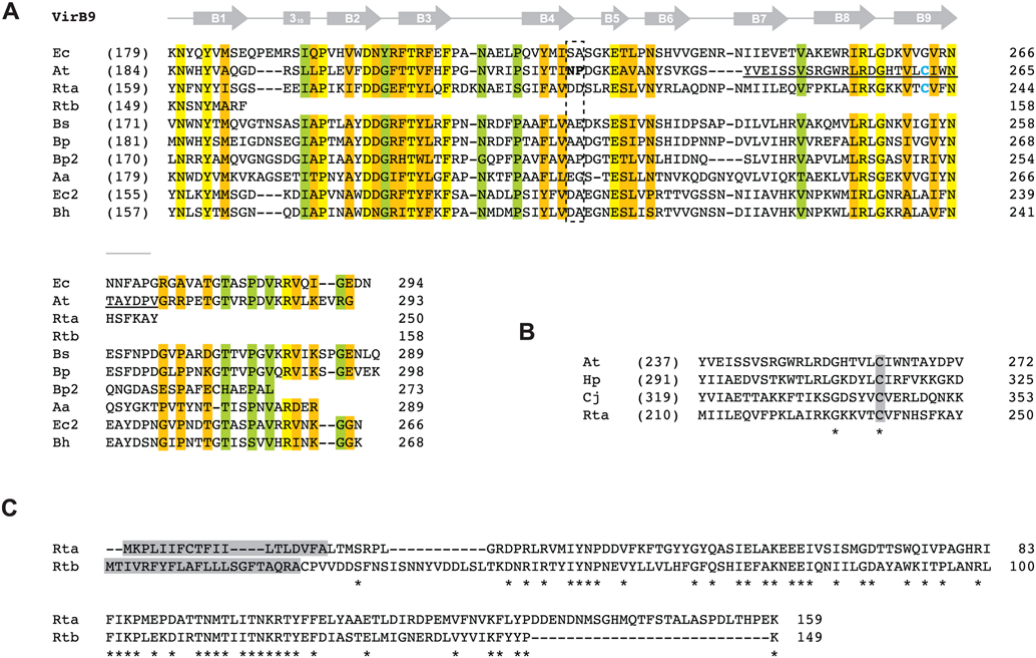
Comparative analysis of VirB9 and VirB9-like proteins with emphasis on two *Rickettsia* orthologs. In all panels, coordinates for each sequence are shown to the right in each block, with numbers in parentheses depicting flanking residues of the alignment not shown. (A) Multiple sequence alignment of ten VirB9 and VirB9-like sequences corresponding to regions of the NMR structure for the VirB9 (TraO^CT^)/VirB7 (TraN) interaction in *Escherichia coli* (IncN plasmid R46) [141]. Secondary structural model is shown above the alignment with arrows (β-strands B1–B9) and a bar (3_10_ helix). Dashed box indicates the region demonstrated to protrude extracellularly from the OM. Blue cysteines in At (C262) and Rta (C241) depict the residues that participate in the disulfide bridge between VirB9_Ti_ and VirB7_Ti_ [53], [54], [116], [135]. Region underlined in VirB9_Ti_ (G242–G271) is depicted in B. Conservation color scheme is the same as in Figure 2 legend, with calculations adjusted for four additional sequences. See text for alignment details. Taxon abbreviations and associated NCBI accession numbers are as follows: Ec = *E. coli* TraO (IncN plasmid R46), NP_511196; At = *Agrobacterium tumefaciens* VirB9, NP_396496; Rta = *Rickettsia typhi* VirB9a, YP_067240; Rtb = *R. typhi* VirB9b, YP_067243; Bs = *Brucella suis* VirB9, NP_699268; Bp = *Bordetella pertussis* VirB9, NP_882293; Bp2 = *Bordetella pertussis* TraK (plasmid pSB102), NP_361041; Aa = *Aggregatibacter actinomycetemcomitans* magB09, NP_067575; Ec2 = *E. coli* TrwF (plasmid pR388), CAA57030; Bh = *Bartonella henselae* TrwF, CAF28337. (B) Illustration of the conserved Cys residue shared between VirB9_Ti_, *R. typhi* VirB9a and the ComB9 proteins of *Helicobacter pylori* (CAA10656) and *Campylobacter jejuni* (NP_863299) [98]. Identical residues are depicted with asterisks and the conserved Cys residue is shaded. (C) Alignment of the N-terminal sequences of VirB9a and VirB9b of *R. typhi*. Identical residues are depicted with asterisks and predicted signal peptides [196], [197] are shaded.

*Rickettsia* genomes contain two VirB9 genes (*virB9a* and *virB9b*) encoded immediately downstream of the two VirB8 genes (**Figure S1**). *virB9a* is more conserved across *Rickettsia* genomes than *virB9b* at the nucleotide (7.6% versus 8.2%) and amino acid (4.7% versus 10.8%) levels. While the entire NTDs of both proteins are comparable to other VirB9 and VirB9-like proteins from other bacteria (data not shown), the CTD of VirB9b is highly truncated and contains residues only in the first β-strand, B1, of the superimposed NMR structure of *E. coli* TraO (a VirB9 analog) (**Figure 5A**). Thus, if at all functional, *Rickettsia* VirB9b proteins lack the necessary domain to interact with VirB7 and VirB7-like lipoproteins. Interestingly, *Rickettsia* VirB9a proteins contain the conserved Cys residue implicated in disulphide bridge formation with VirB7 (discussed below), despite a lack of conservation of the Cys residue in most other VirB9 proteins. This Cys residue is also conserved in ComB9 proteins of *H. pylori* and *C. jejuni* [98] (**Figure 5B**), perhaps lending insight into the origin of the *Rickettsia* T4SS. Finally, despite its highly truncated nature, VirB9b shares mild similarity with VirB9a in the NTD, and both proteins are predicted to contain signal peptides (**Figure 5C**), with the VirB9b signal peptide of *R. typhi* recently confirmed to mediate secretion in an *E. coli*-based alkaline phosphatase gene fusion system [99].

The *Rickettsia* VirB10 gene is conserved across 13 genomes (11% div.), and the corresponding amino acid sequence (10.5% div.) is highly conserved when compared across related proteins from divergent pathogenic bacteria, particularly within the periplasmic CTD for which a crystal has been solved for *H. pylori* ComB10 [95] (**Figure S5**). Like many other (but not all) VirB10 and VirB10-like proteins [100], the *Rickettsia* sequences contain a proline-rich tract in the periplasmic region of the NTD that is predicted to form an α-helical coiled-coil [98], which could mediate oligomerization [95]. Interestingly, the *Rickettsia* sequences contain unique insertions flanking α-helix A3 on both the N- (73 aa) and C- (10 aa) terminal sides. The effects these insertions have on the structure/function of *Rickettsia* VirB10 remain to be determined.

#### Attachment (VirB3)

Like many other bacteria harboring T4SSs, *Rickettsia* contain a VirB3 gene that encodes a small protein (∼95 aa) with a predicted signal peptide and a hydrophobic N-terminal region (**Figure S6**). Many of the hydrophobic residues map to predicted TMS regions, which are characteristic of all sampled VirB3 proteins, and two motifs within the N-terminal region, “(GAT)L(ST)RP” and “GV”, comprise the most conserved sequences of these proteins. Such an occurrence of conserved motifs within predicted signal peptides with such close proximity to TMS regions is unusual, and coupled with a growing number of *virB3*/*virB4* fusions identified from other T4SSs, raises the possibility that VirB3 has more of an IM-periplasmic function [101]. Few other residues are conserved across the sampled taxa. The gene is highly conserved at the nucleotide (6.2%) and amino acid (4%) level across *Rickettsia*, suggesting an essential function for this poorly characterized protein.

### Bioinformatic prediction of three additional scaffold components

Our previous phylogenomic study on *Rickettsia* collectively suggested through consensus genome annotation that, relative to the *A. tumefaciens* archetype T4SS, *Rickettsia* genomes are devoid of Vir scaffold genes encoding VirB1, VirB2, VirB5 and VirB7 [81]. Below, we discuss the various methods used to uncover a *Rickettsia* T4SS that is only lacking a *virB5* homolog. We provide background information for these genes, as they may be prone to misidentification via standard genome automation methods for a variety of reasons discussed below.

#### virB1

VirB1 and related proteins belong to a class of soluble lytic transglycosylases (LTs), enzymes involved in maintaining the integrity of the bacterial cell wall [102]. Specifically, LTs manage the turnover of the murein layer by degrading peptidoglycan, working in concert with murein synthesizing enzymes in a housekeeping fashion [103], [104], [105]. LTs are part of an ancient glycohydrolase superfamily that includes plant chitinases, bacterial chitosanases, goose and hen g-type lysozymes, and phage T4 lysozymes [106]. The specific class of LTs encompassing VirB1 and VirB1-like proteins also includes proteins from other secretion systems and related enzymes present in bacteriophages [66], [67], [107], [108], [109]. This class of “specialized” LTs [110] differs from housekeeping LTs in that peptidoglycan is *locally* disrupted for specific purposes [107], and in the case of VirB1 and related proteins, this purpose is to accommodate the assembly of the T4SS apparatus across the entire cell envelope [66]. Whereas all other Vir components in *A. tumefaciens* have been shown to be essential for type IV secretion, VirB1 is not, as deletion/mutation of *virB1* greatly reduces virulence but does not eliminate tumor formation [42], [111], [112], [113]. VirB1-like proteins are also non-essential in other systems [66], [114], although a VirB1 homolog (HP0523) in the *cag* T4SS of *H. pylori* is critical for both CagA translocation/phosphorylation and interleukin-8 induction in host cells [115]. Aside from self-oligomerization, VirB1 interacts with several other Vir components, namely VirB4 and VirB8-VirB11 [116], [117], illustrating its periplasmic role in peptidoglycan degradation. Apart from the LT domain of VirB1_Ti_, the C-terminal VirB1* is released by cleavage in the periplasm [65] and acts independent of VirB1_Ti_ in tumorigenesis [118]. Specifically, VirB1* interacts with VirB2_Ti_ and VirB5_Ti_ to promote T pilus formation [119]. Despite the conserved consensus sequence “(YH)Vx(KRQ)V” near the cleavage site in VirB1_Ti_, such processing has not been demonstrated in any other VirB1 or VirB1-like sequence to date, although a cleaved VirB1 product was reported in the cell lysate of *Brucella abortus* [113].

**Figure 6 pone-0004833-g006:**
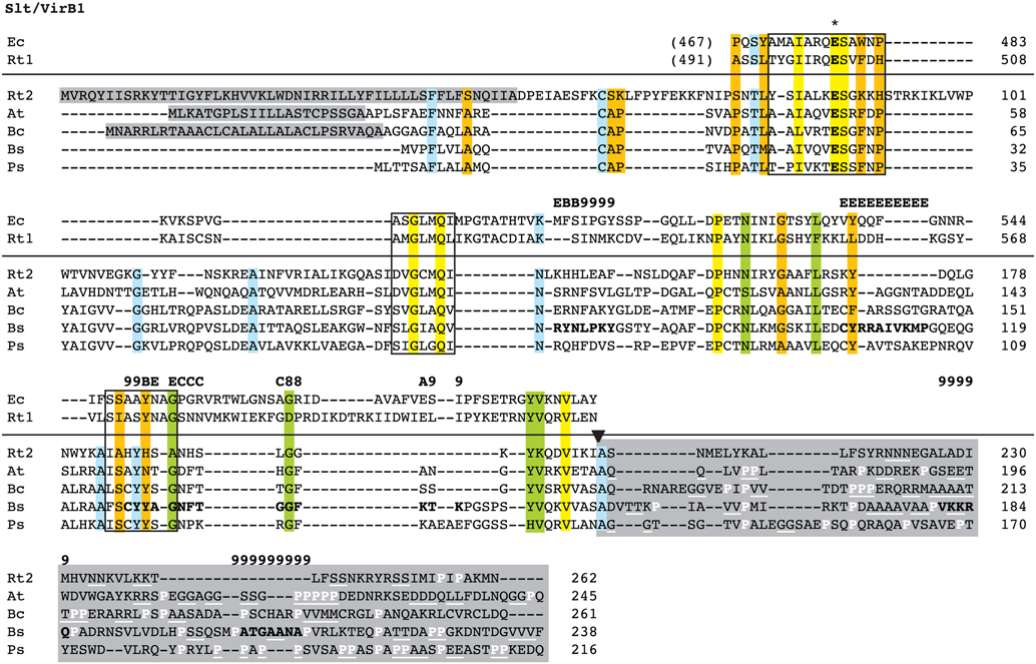
Comparison of soluble lytic transglycosylase (LT) domains of two rickettsial proteins to the LT domain of *Escherichia coli* Slt70 and “specialized” LT domains within VirB1 proteins from four pathogenic bacteria. Alignment below horizontal line of entire VirB1 and VirB1-like sequences performed using MUSCLE [194], [195] (see text). Above the horizontal line is the alignment of the LT domains of *E. coli* Slt70 and the larger rickettsial sequence using EMBOSS (Needle algorithm). This alignment was fitted to the alignment below the horizontal line based on a second needle alignment for *E. coli* Slt70 and the VirB1 protein from *Brucella suis* [116]. Conserved residues previously implicated in enzymatic activity are within boxes [210]. An asterisk depicts the essential active-site Glu [67], [120], [211], [212]. Interactions between VirB1 and other T4SS components as revealed by peptide array mapping [65] are shown over the alignment with the following symbols: 8 = VirB8; 9 = VirB9; E = VirB11; A = VirB8+VirB9; B = VirB9+VB11; C = VirB8+VirB11. Conservation color scheme is the same as in Figure 2 legend, except orange = invariant over 70% of sequences and blue = invariant across all VirB1 and VirB1-like sequences, with conserved contrasting residues in the LT domain alignment also colored blue (if applicable). Conservation was not assessed for columns including gaps. Black arrow depicts the cleavage site of *Agrobacterium tumefaciens* VirB1 protein [65] (NOTE: cleavage in other VirB1 and VirB1-like proteins has not been definitely demonstrated). Entire CTDs are shaded and Pro residues are colored white and di- and poly-repeat residues underscored white. Shaded N-terminal residues depict signal peptides predicted by SignalP 3.0 [196]. Taxon abbreviations and associated NCBI accession numbers are as follows: Ec = *E. coli* Slt70, P0AGC3; Rt1 = *Rickettsia typhi* putative transglycosylase, YP_067347; Rt2 = *R. typhi* putative soluble lytic murein transglycosylase, YP_067402; At = *A. tumefaciens* VirB1, NP_053381; Bc = *Burkholderia cepacia* genomovar III VirB1, AAK50141; Bs = *B. suis* VirB1, AAN33281; Ps = *Pseudomonas syringae* VirB1, AAR02172.

Searches of the *Rickettsia* database [80] for genes annotated as coding for LTs yielded two conserved families. The first family contained genes annotated as either “soluble lytic murein transglycoslyase” or “hypothetical protein”. This gene family encodes proteins of an average length 650 aa, consistent with the size of the product of *E. coli slt*. Considerable variation in annotation existed for the second gene family, ranging from “hypothetical protein” to “invasion protein” to “soluble lytic transglycosylase”. These genes encode proteins averaging 290 aa, a size typical of many specialized LTs. Interestingly, one member of this family, *R. prowazekii* ORF 457, was previously suggested as a possible candidate for VirB1 in a comparison of several divergent T4SSs [26]. This prediction was never incorporated into genome annotation. A search of the NCBI protein database for “VirB1” yielded 142 results, of which all results containing “VirB1” in the protein annotation were selected for comparison with the smaller rickettsial LT domain containing proteins. These proteins were aligned and a subset of this alignment illustrates the limited conservation in VirB1 and VirB1-like sequences across divergent bacteria (**Figure 6**). Collectively, the selected sequences are most conserved in the regions corresponding to the active site motifs of the crystal structure of *E. coli* Slt [120], which are also conserved in the larger *Rickettsia* “soluble lytic murein transglycoslyase” family. The C-terminal region of VirB1* processing is also conserved as previously reported [119], and all predicted VirB1* sequences contain tracts of repeated residues, particularly Pro rich tracts. Across the 13 *Rickettsia* genomes, these putative VirB1 sequences exhibit the least conservation of all Vir components at the nucleotide (13.9%) and amino acid (16.3%) level (after the variable flanking sequences of VirB6 proteins are excluded, see above). Accordingly, we suggest that *Rickettsia* genomes contain both housekeeping LTs orthologous to *E. coli* Slt and related Slts, as well as specialized LTs similar to VirB1, which are likely to play a role in type IV secretion via the localized degradation of peptidoglycan.

#### virB2

VirB2 and related proteins comprise the major pilin subunit of the T pilus [121], [122] and are also essential components of the mating channel [42], [62], [123], [124]. The signal sequences of these proteins are cleaved in the periplasm, followed by additional species-specific processing of the N- and C-termini [92], [125], [126], [127], [128], [129], [130]. In *A. tumefaciens*, VirB2_Ti_ is made circular through a cyclization process mediated by an uncharacterized chromosomal-encoded gene product [126], [127]. Various laboratory strategies have identified VirB2 in complexes with VirB5 and VirB7 [55], [131], [132], [133], [134], [135], supporting its role in T pilus formation. The VirB2-VirB5 pilus complex is dependent on the periplasmic interaction between VirB4 and VirB8, which results in the formation of extracellular pili [135]. However, all VirB2 and VirB2-like proteins are incorporated into the IM [2], as evident by at least two predicted TMS regions in all analyzed sequences. Polymers [134] of the protein are critical for substrate transfer in the mating channel [124], [136] and possibly span the entire periplasm [101]. Given the recent hypothesis that T4SSs have dual (and independent) biogenesis roles in T pilus formation and substrate transfer [101], it is apparent that VirB2 and VirB2-like proteins are essential for both processes [2].

**Figure 7 pone-0004833-g007:**
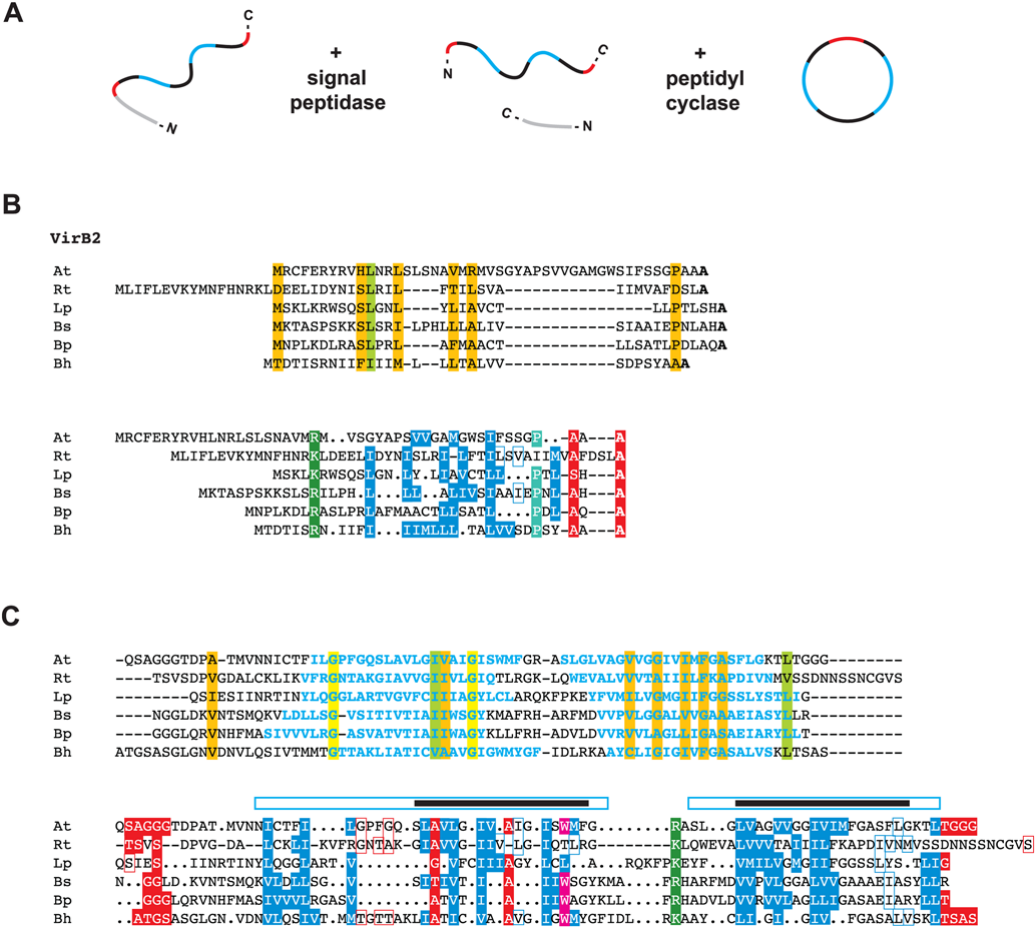
Characteristics and comparative analysis of VirB2 and VirB2-like sequences from six divergent bacterial species. (A) Schema illustrating the processing (cleavage and cyclization) of *Agrobacterium tumefaciens* VirB2 [125], [126], [127]. (B, C) Automated (top) and manual (bottom) alignment of VirB2 and VirB2-like proteins across six bacterial species. For automated alignment, conservation color scheme is the same as in Figure 2 legend. See text for alignment details. Manual alignment shows a putative *Rickettsia* VirB2 protein threaded to a prior homology model [122]. Boxes with colored borders depict further support for this model with the addition of the rickettsial sequence. Gaps (versus dots) depict adjustments to the original model. Taxon abbreviations and associated NCBI accession numbers are as follows: At = *A. tumefaciens* VirB2, AAB28331; Rt = *Rickettsia typhi* hypothetical protein, YP_067147; Lp = *Legionella pneumophila* lvhB2, CAB60051; Bs = *Brucella melitensis suis* VirB2, ABM66830; Bp = *Bordetella pertussis* pertussis toxin transport protein, NP_882287; Bh = *Bartonella henselae* VirB2, AAD48919. (B) Predicted signal peptides based on Signal P v.3.0 output [196] (top) and laboratory-defined processed sites [122] (bottom). Bolded residues (top) depict conserved Ala residues implicated by both methods as the cleavage site. (C) Core regions of the processed VirB2 and VirB2-like proteins. Blue residues (top) depict predicted TMS regions [93], and these regions are enclosed over the predicted TMS regions (black bars) in the prior homology model [122].

VirB2 proteins were previously identified in only two *Rickettsia* genomes, *R. canadensis* and *R. bellii* str. OSU 85-389. Thus, our recent generation of orthologous groups (OGs) using consensus annotation across 10 genomes labeled this family as a conserved hypothetical protein [81]. A search of the NCBI protein database for “VirB2” yielded 636 results, of which the top 50 were selected for comparison with the rickettsial putative VirB2 proteins. A subset of this comparison illustrates the limited conservation across related VirB2 and VirB2-like proteins (**Figure 7**). Both automated alignment and manual alignment based on a prior homology model for VirB2 [122] support a conserved Ala residue at the predicted signal peptide cleavage site, as well as two hydrophobic TMS regions. Our automated alignment corroborated a previous study that reported several (unspecified) conserved Gly residues across a VirB2 alignment [101], as two conserved Gly residues were recovered within the N-terminal TMS region. However, these conserved Gly residues were not predicted in the prior homology model [122] or in a more recent comparison of diverse VirB2 and VirB2-like proteins [137]. Across the 13 *Rickettsia* genomes, the putative VirB2 sequences are conserved at the nucleotide (10.5%) and amino acid (11.6%) level, suggesting these proteins function in substrate transfer through the mating channel.

#### virB7

VirB7 and related proteins are typically the smallest components of T4SS scaffolds and are seemingly not present within all identified systems [18]. However, the extremely small size of these lipoproteins poses a challenge for detection with blast, thus leaving open the possibility that VirB7 and related lipoproteins have yet to be characterized within some T4SSs. VirB7 is secreted to the periplasm [58], [138] and interacts with the CTD of VirB9, stabilizing the OM-associated protein [53], [54], [58], [59], [139] and other Vir components in the mating channel [45], [131]. As discussed above, the critical disulphide bond linking VirB7_Ti_ and VirB9_Ti_ [53], [54], [116], [135] is not conserved in most other T4SSs, suggesting other protein interactions suffice in heterodimer stabilization [140]. VirB7_Ti_ is also IM-associated [58] as well as found in the extracellular milieu in abundant levels [128], even independent of other VirB protein synthesis [101]. VirB7_Ti_ forms homodimers via intermolecular disulphide bonds, and also interacts with VirB2 and VirB5 [128], [132], [134]. Dimerization in other VirB7 proteins via Cys-Cys bridges should be possible given the highly conserved nature of the lipo-processing site (Cys-24 of VirB7_Ti_).

**Figure 8:**
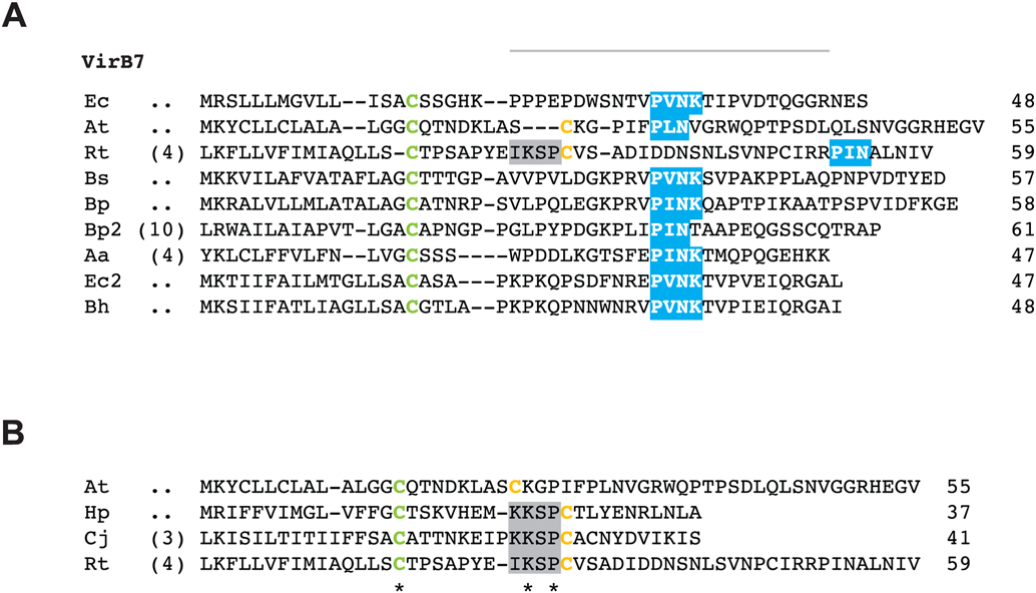
Comparative analysis of VirB7 and VirB7-like lipoproteins with emphasis on the similarities between *Rickettsia* VirB7 and ComB7 proteins. In both panels, coordinates for each sequence are shown to the right, with numbers in parentheses depicting flanking residues of the alignment not shown. Green Cys = predicted lipoprotein cleavage site [197] and orange Cys = residue in disulphide bridge with VirB9/VirB9-like proteins. The “[KI]KSP” motif shared by *Rickettsia* spp. VirB7 proteins and ComB7 proteins from *Helicobacter pylori* and *Campylobacter jejuni* is shaded gray. See text for alignment details. (A) Multiple sequence alignment of nine VirB7 and VirB7-like sequences. Gray bar depicts region of the NMR structure for the VirB9 (TraO^CT^)/VirB7 (TraN) interaction in *Escherichia coli* (IncN plasmid R46), with the conserved “P[ILV]NK” motif in blue [141]. Taxon abbreviations and associated NCBI accession numbers are as follows: Ec = *E. coli* TraN of IncN plasmid R46, NP_511194; At = *Agrobacterium tumefaciens* VirB7, NP_536291; Rt = *R. typhi* hypothetical protein, YP_067241; Bs = *Brucella suis* VirB7, AAN33275; Bp = *Bordetella pertussis* TraI protein of plasmid pSB102, NP_361043; Bp2 = *B. pertussis* putative bacterial secretion system protein, NP_882291; Aa = *Aggregatibacter actinomycetemcomitans* lipoprotein, NP_067577; Ec = *E. coli* TrwH protein, FAA00034; *Bartonella henselae* TrwH-like protein, AAM82208. (B) Multiple sequence alignment of VirB7_Ti_, *R. typhi* putative VirB7 and the ComB proteins of *H. pylori* (CAA10654) and *C. jejuni* (NP_863349). Invariant residues are denoted with an asterisk under the alignment.

In a comparison of several divergent T4SSs, *R. prowazekii* ORF 288 was suggested as a possible candidate for VirB7 [27], a conclusion likely reached based on synteny, as the gene for RP288 is located directly upstream of *virB8b* (**Figure S1**). However, this ORF is not annotated as VirB7 in any *Rickettsia* genome [80], [81], a likely consequence of its small size and zero blastp hits to related sequences from other bacteria. We took RP288 and orthologous proteins from the additional 12 *Rickettsia* genomes and aligned them to 70 VirB7 and VirB7-like proteins retrieved from GenBank, a subset of which reveals the limited sequence conservation in this protein family (**Figure 8**). The Cys lipoprotein-processing site was invariant across all 82 proteins in the larger alignment, however the remaining C-terminal region was poorly aligned with a high rate of length heterogeneity. In the smaller alignment, manual adjustment was made to the C-terminal region around the conserved “P[ILV]NK” motif [141], and the Cys residues of VirB7_Ti_ and *Rickettsia* VirB7 were homologized (**Figure 8A**). Unless using excessive gap insertion, both landmark features could not be homologized across the alignment, as the *Rickettsia* protein has an extended sequence between the Cys residue and the P[ILV]NK motif. In the TraO^CT^/TraN NMR structure of the *E. coli* pKM101 T4SS, TraN (VirB7) winds around part of the β-sandwich formed by TraO (VirB9), with the P[ILV]NK motif of TraN embedded within a deep hydrophobic pocket of TraO [141]. The authors suggested the distance from the lipidated Cys to this conserved motif (∼16 aa) could be critical in orienting TraO in the OM. The effect the longer sequence in *Rickettsia* VirB7 proteins has on a potential VirB7/VirB9 interaction is unknown, but given a predicted lipo-processing site, a second conserved Cys residue, a potential P[ILV]NK motif, and a conserved genomic position immediately upstream of *virB8b*, we suggest these small ORFs are strong candidates for VirB7 proteins. Interestingly, a manual alignment of VirB7_Ti_ with the putative *Rickettsia* VirB7 and the ComB7 proteins of *H. pylori* and *C. jejuni* revealed a conserved motif “[KI]KSP” directly flanking the second conserved Cys in the latter three taxa (**Figure 8B**). Perhaps this feature accommodates the extra length in the *Rickettsia* sequences, and at very least presents the possibility that the VirB7/VirB9 structural interaction is flexible and variable across different T4SSs.

### *Rickettsia* T4SS: an evolutionarily conserved archipelago

**Figure 9:**
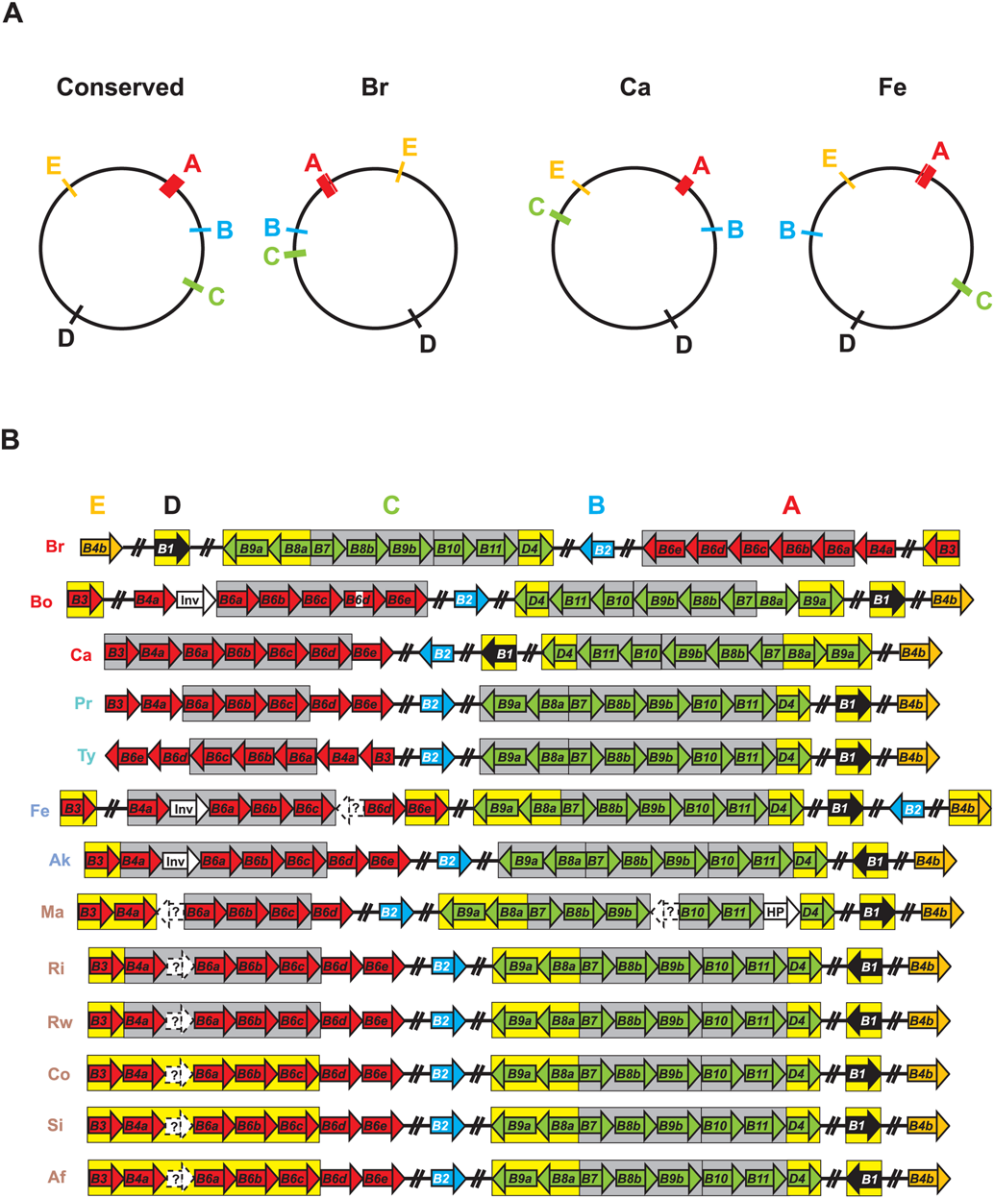
Structure of the *Rickettsia* T4SS archipelago garnered from the comparison of 13 genomes. Vir genes are grouped into five islets as follows: red = islet A (*virB3*, *virB4a*, *virB6a*-*virB6e*), blue = islet B (*virB2*), green = islet C (*virB9a*, *virB8a*, *virB7*, *virB8b*, *virB9b*, *virB10*, *virB11*, *virD4*), black = islet D (*virB1*), and orange = islet E (*virB4b*). (A) Illustration of the conserved genomic locale of the *vir* T4SS genes (left), as well as minor deviations in the genomes of *R. bellii* (str. RML 369-C), *R. canadensis* and *R. felis*. (B) Schema illustrating the composition of the five islets across 13 genomes. Genes are not drawn to scale (see Table 1 and Figure S1 for approximate ORF lengths). White ORFs depict non-Vir genes nestled between Vir genes: HP = hypothetical protein, Inv = invasion protein (Note: a fourth protein within this orthologous group from *R. conorii* is not found within the T4SS archipelago). ORFs within predicted operons [143] are enclosed in boxes: gray = strictly *vir* operons, yellow = *vir* operons containing non-Vir genes (NB: see Table 2 for non-Vir genes associated within *vir* operons). Genes predicted by fgenesb but not PATRIC are within dashed white boxes and annotated as “?”. Genome codes are as follows: Br = *R. bellii* str. RML369-C, Bo = *R. bellii* str. OSU 85 389, Ca = *R. canadensis* str. McKiel, Pr = *R. prowazekii* str. Madrid E, Ty = *R. typhi* str. Wilmington, Fe = *R. felis* str. URRWXCal2, Ak = *R. akari* str. Hartford, Ma = *R. massilae* str. MTU5, Ri = *R. rickettsii* str. Sheila Smith CWPP, Rw = *R. rickettsii* str. Iowa, Co = *R. conorii* str. Malish 7, Si = *R. sibirica* str. 246, and *R. africae* str. ESF-5. The genome codes are colored to reflect classification [81], [147]: red = ancestral group rickettsiae (AG), turquoise = typhus group rickettsiae (TG), blue = transitional group rickettsiae (TRG), and brown = spotted fever group rickettsiae (SFG).

Whether encoded on plasmids (common) or chromosomes (rare), the components of T4SSs are typically arranged in one operon or several adjacent operons [101], [142]. Regarding T4SSs closely related to the *vir* system, genes encoding T4CPs (when present) are commonly not arrayed with VirB and VirB-like genes, but located in nearby operons that often include additional genes related to substrate processing [142]. Collectively, the 18 Vir genes within *Rickettsia* genomes are scattered throughout the circular chromosomes, with the T4CP gene (*virD4*) arrayed *with* other Vir components (**Figure 9A**). Given the conserved synteny in *Rickettsia* genomes, especially in regards to the three derived groups [81], the various Vir components, now expanded to five genomic regions in most cases, can be organized into five “islets” (A–E) comprising an archipelago that spans the entire genome of each taxon. Ten of the 13 analyzed genomes have the five islets in similar locations, with islets C and D in *R. canadensis* and islet B in *R. felis* within regions of rearrangement unique to both genomes. The large rearrangement of most of the *R. bellii* str. RML369-C genome relative to all other *Rickettsia* genomes positions its T4SS in a mirror image relative to the conserved arrangement.

Operon prediction [143] within these islets was variable across the 13 genomes, and several islets were predicted to contain additional genes outside of those encoding the T4SS scaffold (**Figure 9B** **,** **Table 2**). Given the propensity for T4SS effectors and regulators to be encoded within close proximity to the scaffold genes [144], these genes will be of interest to future studies that aim to identify protein substrates (if any) specific to this transporter, as well as factors that regulate its expression. Two genes in particular are interesting in part due to their presence in all 13 genomes. *gppA* encodes an enzyme similar to RelA/SpoT homologs that functions as a mediator of the stringent response, hence coordinating a range of cellular activities in reaction to changes in nutritional abundance [145]. Given that expression of the T4SS of *Brucella* spp. has been demonstrated to be *gppA-*like dependent [146], this protein is a potential candidate for regulation of the *Rickettsia* T4SS. The second conserved non-Vir gene encodes a conserved hypothetical protein within the predicted VirB1 operon, an ideal location for an effector protein given the role of VirB1 in murein degradation. blastp results using this protein as a query showed little similarity to other proteins (data not shown). *In silico* characteristics, such as lack of predicted signal peptides and a skew in positively charged residues in the C-terminal region, a common attribute of some T4SS effectors [2], make this curious protein a candidate for type IV secretion. Interestingly, the protein from *R. sibirica* bound VirB8 in a bacterial two-hybrid screen [74]. Finally, several of the remaining non-Vir genes within predicted *vir* operons are attractive and open avenues for exploring lineage specific virulence factors associated with the *Rickettsia* T4SS.

**Table 2:** Non-Vir Genes Within Predicted Operons Encoding the *Rickettsia* T4SS.

Islet^1^	*vir* 2	Associated non-Vir genes^3^	Distribution^4^
A	*B3*	*argB*, acetylglutamate kinase	Br, Bo, Fe, Ak, Ri, Rw
		CHP, 95 aa	Br, Bo, Fe, Ak, Ri
		GTP-binding CHP, 212 aa	Fe, Ak, Ri, Rw
A	*B3*-*B4a*	*argB*, acetylglutamate kinase	Ma
		GTP-binding CHP, 213 aa	Ma
A	*B3*-*B6c*	putative ORF (fgenesb), 61 aa	Af
		*argB*, acetylglutamate kinase	Co, Si, Af
		GTP-binding CHP, 213 aa	Co, Si, Af
		putative ORF (fgenesb), 56 aa	Co, Si, Af
A	*B6e*	*vapB*, antitoxin of VapB/VapC system	Fe
		*vapC2*, COG1487: PIN domain protein	Fe
C	*B8a*-*B9a*	*mrpD*, Na(+)/H(+) antiporter subunit D	Co, Si, Af
		*mrpC*, Na(+)/H(+) antiporter subunit C	Br, Ca, Fe, all SFG genomes
		putative ORF (fgenesb), 49 aa	Fe
C	*B9a*	*mrpC*, Na(+)/H(+) antiporter subunit C	Bo
C	*D4*	*gppA*, GTP dp pyrophosphatase	All 13 genomes
		*vapB1*, antitoxin of VapB/VapC system	Br, Bo
		*vapC1*, toxin of VapB/VapC system	Br, Bo
		*ndhF*, NAD(P)H dehydrogenase 5	Br
		ptrB, protease 2	Br, Bo
D	*B1*	COG0536, predicted GTPase	Br
		putative ORF (fgenesb), 84 aa	Bo
		putative ORF (fgenesb), 72 aa	Bo
		HP, 101 aa	Fe
		CHP, ∼171 aa	All 13 genomes
		HP, ∼130	Br, Fe
		rickettsial palindromic element	Fe
E	*B4b*	HP, 74 aa	Fe

1 Corresponding to islets A (*virB3*-*virB6e*), C (*virB9a*-*virD4*), D (*virB1*), E (*virB4b*) (See **Figure 9**).

2 According to operon prediction by fgenesb [143].

3 Consensus annotation from PATRIC [80] or fgenesb prediction. HP = hypothetical protein, CHP = conserved hypothetical protein.

4 Taxa included within operon prediction. Underlined taxa depict split ORFs.

**Figure 10:**
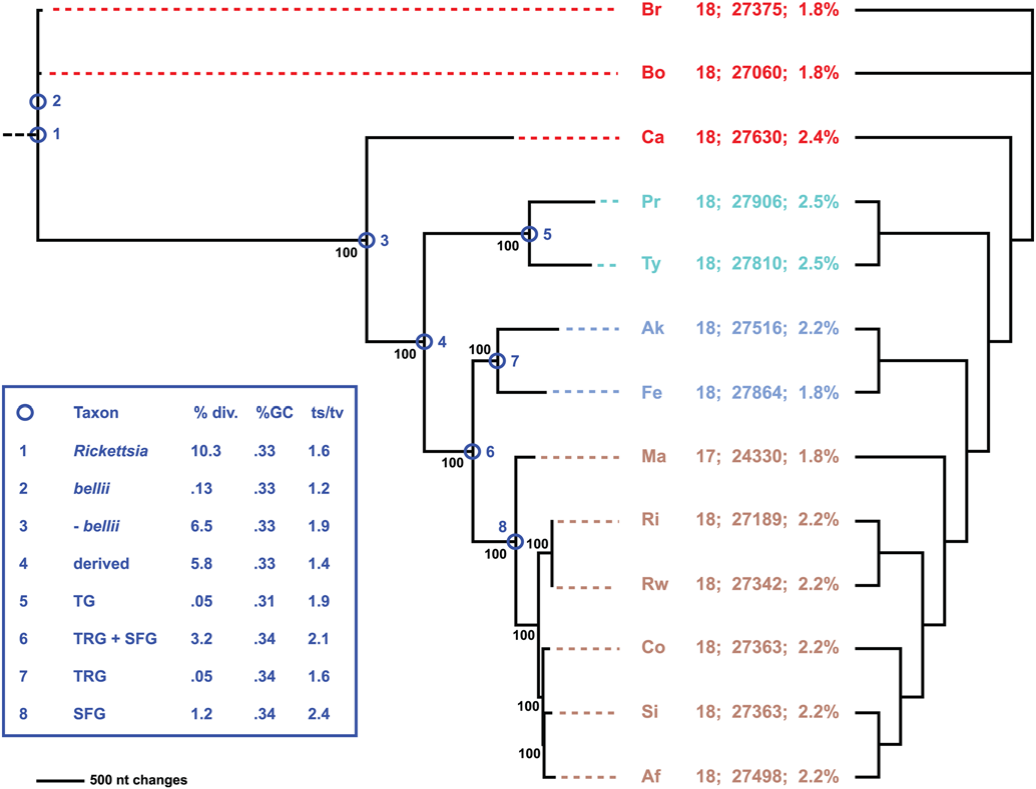
Phylogeny estimation of 18 putative *vir* genes of the *Rickettsia* T4SS. Single most parsimonious tree of 12716 steps (6858 parsimonious characters of 28554 total characters). Branch support is from 1 million bootstrap replications. Taxon codes and coloring scheme as described in the Figure 9 legend. Statistics to the right of taxon codes: number of T4SS genes; number of nts encoding T4SS; percentage of genome encoding T4SS. Cladogram on right depicts phylogeny of core orthologous groups (proteins) [80]. Inset shows statistics computed for eight nodes of the tree, following our classification scheme [81].

Combined phylogeny estimation from all 18 components of the *Rickettsia* T4SS corroborated the species tree generated from an analysis of over 700 core *Rickettsia* genes (**Figure 10** **, Figure S7)**. The inclusion of additional genomes released subsequent to our prior analyses, namely *R. massiliae*, *R. africae* and *R. rickettsii* str. Iowa, does not overturn our tree-based classification of *Rickettsia* into four major groups: ancestral group (AG), typhus group (TG), transitional group (TRG), and spotted fever group (SFG) rickettsiae [81], [147]. All but one of the phylogeny estimations of the independent Vir components failed to recover the species tree topology (**Table 3** **; Figure S8**), however deviations were minor and can be explained by the properties of the data (too few informative sites or homoplasy). In these latter cases, either the independent analyses failed to resolve the highly similar derived members of the SFG rickettsiae (*R. rickettsii*, *R. conorii*, *R. sibirica*, *R. africae*), for which the assignment of species names has received recent criticism [82], or failed to place *R. canadensis* as basal to the TG, TRG and SFG rickettsiae. We previously reported that the fluctuating position of *R. canadensis* was dependant upon the analyzed gene(s) and phylogenetic utility of the said gene(s) [81], and our current analysis, as well as a very recent study [148], further supports this phenomenon. Altogether, phylogeny estimation supports a single inheritance of the 18 Vir components from the *Rickettsia* ancestor, with one gene loss (*virB6e* in *R. massiliae*), one split gene (*virB6d* in *R. bellii* str. OSU 85 389), three major gene rearrangements (**Figure 9A**), and several minor switches of coding strand (**Figure 9B**) the defining diversifying factors of this conserved system. Our analysis corroborates a recent study that analyzed a subset of Vir components (VirB3, VirB4, VirB8, VirB8, VirB11) from 31 taxa of *Alphaproteobacteria* and demonstrated vertical inheritance of Vir genes across the entire Rickettsiales [149].

**Table 3:** Summary Statistics for the Vir Genes Encoding the *Rickettsia* T4SS.

Vir	aa seqs			nt seqs					
	div^1^	PI^2^	Tree^3^	div^1^	PI^2^	Tree^3^	GC^4^	ts/tv^5^	dN/dS^6^
D4	4.2	54	N	8.1	329	N	0.35	1.9	0.06
B4a	3.7	72	N	6.7	380	N	0.34	2.5	0.05
B4b	9.9	195	N	9.2	570	N	0.28	1.9	0.13
B11	4.8	33	N	9.7	186	N	0.37	1.9	0.06
B6a	14.7	374	N	12.8	1054	N	0.36	1.5	0.23
B6b	13.2	196	N	11.2	565	N	0.34	1.9	0.2
B6c	15.2	285	N	13.2	848	N	0.35	1.2	0.27
B6d	12.9	263	N	10.3	682	N	0.32	1.9	0.18
B6e	12.4	308	N	11.1	871	N	0.33	1.6	0.17
B8a	13.4	63	Y	10	163	N	0.26	1.7	0.26
B8b	9.4	55	N	9	161	N	0.31	1.3	0.13
B9a	4.7	27	N	7.6	130	N	0.35	1.8	0.06
B9b	10.8	43	N	8.2	98	N	0.26	2.5	0.14
B10	10.5	108	N	11	356	N	0.38	1.4	0.14
B3	4	8	N	6.2	43	N	0.31	1.4	0.1
B1	16.3	93	N	13.9	271	N	0.32	1.3	0.6
B2	11.6	35	N	10.5	104	N	0.32	1.9	0.26
B7	15.3	23	N	11	47	N	0.32	1.4	0.21

1 Avg. pairwise differences across all sequences divided by avg. sequence length.

2 Number of parsimony informative characters.

3 Tree corroborates (Y) or disagrees with (N) species tree. All trees are shown in **Figure S4**.

4 %GC of all codon positions.

5 Ratio of transitions to transversions.

6 Ratio of non-synonymous to synonymous substitutions.

### Lateral acquisition of Rickettsiales T4SS

Our synteny and phylogenetic analyses of the *Rickettsia* T4SS yield results consistent with previous studies on the T4SS operon structure of the closely related rickettsiae *Anaplasma*, *Ehrlichia* and *Wolbachia*, as these genomes have at least two *vir* clusters (*virB3*-*virB4*-*virB6* and *virB8*-*virB9*-*virB10*-*virB11*-*virD4*) in well-separated regions of their genomes, often with duplications of the VirB4, VirB6, VirB8, and VirB9 components [149], [150], [151], [152]. Interestingly, the genome sequence of the sister taxon to *Rickettsia*, *Orientia tsutsugamushi*, revealed an unprecedented degree of Vir-like gene duplication (mostly comprising components of the *E. coli tra* operon), with 359 ORFs putatively coding for various components of a T4SS throughout 79 sites in the genome [153]. Thus a remarkable array of diverse T4SSs exists across species in the Rickettsiales, yet intrageneric conservation of these systems is apparently high. Interestingly, archipelagos of *vir* islets seem to be characteristic of all genomes in the Rickettsiales, and it has been shown in one system (*Ehrlichia chaffeensis*) that the same protein, EcxR, regulates all of its *vir* islets [150]. A search for a protein orthologous to EcxR in all of the *Rickettsia* genomes proved futile, although the small size of this protein (108 aa) is likely confounding blastp searches. It is expected that similar T4SS regulators will be identified in other Rickettsiales genomes, given the need to tightly regulate all of the scaffold components for efficient transporter function.

Collectively, conserved features across rickettsial T4SSs hint at a single inheritance that possibly fostered the route to obligate intracellular symbiosis (mitochondria, symbiotic rickettsiae) and subsequent pathogenesis after the divergence of the rickettsial ancestor from its free-living marine relative *Pelagibacter ubique* [86]. Given that phylogeny estimation [18] and protein homology network-based clustering [29] of T4SSs imply the Rickettsiales *vir* system is closely related to the T4SSs from certain γ- (*Legionella* spp. and *Photobacterium profundum*) and ε-proteobacteria (*H. pylori*, *Wolinella succinogenes* and *C. jejuni*), it is likely ancestors of these distantly related organisms resided in a common environment and acquired similar T4SS genes from one or several progenitors. Several lines of evidence support this. First, *Rickettsia bellii* str. RML369-C [78], *Legionella pneumophila* [154] and *H. pylori* [155] are all capable of growth in various species of amoeba. Given the role of protozoa as reservoirs for amoeba-resistant organisms [156], [157], [158], it is likely amoeba provided a breeding ground for rickettsial species and distantly related microbes [78]. Second, some members of the third class of Rickettsiales, the Holosporaceae, are also found in amoeba [159], [160], [161]. This suggests at least two lineages branching from the Rickettsiales ancestor were capable of endosymbiosis within nucleated single-celled organisms (the mitochondrial endosymbiosis [162] being the other). Third, a gene encoding a Sec7-domain-containing protein, RalF, is known in prokaryotes only from *Rickettsia* and *Legionella* spp. [163]. In *Legionella*, RalF is a T4SS (Note: of the *dot*/*icm* type B T4SS) effector that functions as a guanine nucleotide exchange factor that recruits ADP-ribosylation factor to occupied phagosomes, permitting *Legionella* to replicate free from the host immune system and subvert vesicular trafficking [7]. While the NTD and associated central Sec-7-capping-domain are conserved in all *Rickettsia* genomes aside from SFG rickettsiae [81], a function for *Rickettsia* RalF or association with the T4SS has still yet to be determined. Finally, the abovementioned similarities between *Rickettsia* VirB7/VirB9a proteins and the ComB7/ComB9 proteins of *H. pylori* and *C. jejuni* may hint at a shared OM anchoring system for the mating channel common to VirB7_Ti_/VirB9_T_, although a similar role in competence (versus protein translocation) in *Rickettsia* cannot be ruled out.

### Vir gene evolution via duplication and intrachromosomal recombination

It is not uncommon for bacteria to harbor multiple unrelated T4SSs; e.g., the *cag* and *comB* systems of *Helicobacter* [164], [165], the *dot*/*icm* and *lvh* systems of *Legionella* [27], [166], [167], and the *vbh*, *virB* and *trw* systems of *Bartonella* [168], [169]. While many T4SSs are mainstays that define bacterial lifestyles, other T4SSs may be strain specific and highly plastic throughout bacterial populations, especially if encoded on plasmids [170]. In *Legionella*, components from the *lvh* system have been shown to complement analogs of the *dot*/*icm* system [27], suggesting functional resilience despite sequence plasticity. Growing studies utilizing cross-species heterologous complementation attest to this functional resilience [171], [172]. Despite this, gene duplications *within* individual T4SSs are not the norm (Seubert et al., 2003; Alsmark et al., 2004), and the functional redundancy is likely best explained on a case-by-case basis. For example, a recent study of the *trw* T4SS across seven *Bartonella* taxa revealed 7–8 copies of *trwL* (*virB3*) and 2–5 copies of the *trwJ*-*H* operon (*virB5*-*7*) [173]. Phylogenetic analysis of the TrwL duplicates revealed probable neofunctionalization within a subset of the duplicates, while the flanking TrwJ and TrwH duplicates of the *trwJ*-*H* operon appeared to be products of LGT speculated to have arisen due to the coevolution of these OM proteins with erythrocyte surface structures.

**Figure 11:**
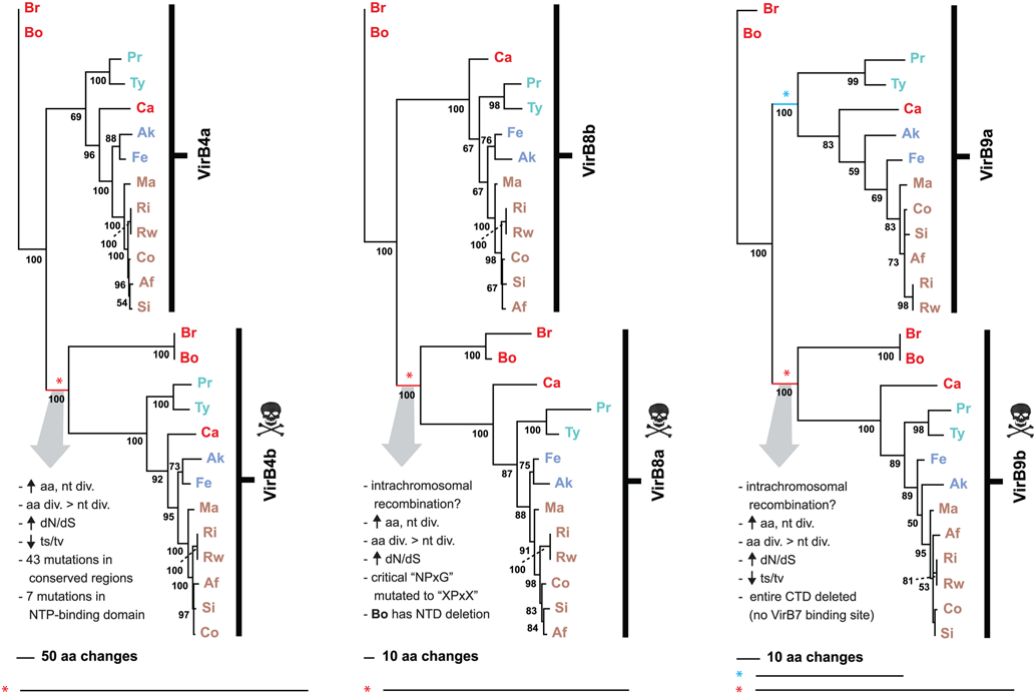
Phylogeny estimation of duplicate VirB4, VirB8 and VirB9 proteins. All trees estimated under parsimony (see text for details). Branch support is from 1,000 bootstrap replications. Taxon codes and coloring scheme as described in the Figure 9 legend. Asterisks depict shortened branches that are shown in their full length below the tree. Skull and crossbones depict lineages leading to probable pseudogenization or neofunctionalization. Characteristics of these divergent sequences are listed.

Our recent generation of *Rickettsia* OGs across 10 [81] and 12 [80] genomes unambiguously delineated the duplicate VirB4, VirB8 and VirB9 and quadruplicate VirB6 genes into discrete OGs. Sequence divergence and other summary statistics (**Table 3**) as well as phylogenetic analysis (**Figure S4,** **Figure 11**) strongly suggest all of these genes were vertically acquired (i.e., the duplications were ancestral). Analysis of the three duplicate components (VirB4, VirB8 and VirB9) suggests pseudogenization or neofunctionalization has occurred in one member from each family, as the suspects (*virB4b*, *virB8a* and *virB9b*) comprise the only three T4SS genes that deviate from the average genomic base composition and drop below 30% GC (**Table 3**). The characteristics of these divergent duplicates (discussed above) are mapped onto individual phylogeny estimations (**Figure 11**). Given that a *virB4* active site mutant stabilized VirB3 and VirB8 and promoted T-pilus formation in *A. tumefaciens* [135], it is possible VirB4b still can function as an IM gate that stabilizes a T4SS channel, facilitating (but not powering) the uptake or release of substrates. A full length VirB8 protein unable to dimerize or properly interact with other Vir components may still function in formation of a separate membrane-spanning channel. Also, a chaperone-assisted process may help VirB8b overcome the critical mutations in the linker between helix α4 and strand β4. However, given the asymmetrical position of the VirB8 and VirB9 duplicates around the putative VirB7 ORF (**Figure 9**), it is probable that these genes have experienced an intrachromosomal recombination event early in their evolution that deleted the CTD of VirB9b. In *Rickettsia*, intrachromosomal recombination has been implicated in the split of the *rrs* and *rrl* loci [174] and has likely shaped the atypical clustering of the Tuf and Fus genes [175]. Thus it is a common phenomenon in *Rickettsia* genomes. While neofunctionalization is difficult to suggest for VirB9b, given the deletion of the entire domain known to interact with VirB7, potential alternative functions for VirB8a and VirB4b are not unrealistic. However, in-frame pseudogenes that are still expressed may be degraded post-transcriptionally given the unusually high amount of genes involved in RNA degradation in *Rickettsia* genomes [71].

None of the proliferated VirB6 genes deviate from the average genomic base composition (**Table 3**), and given the conservation of the essential Trp residue in all sequences, as well as the constraints on hydrophobicity throughout large portions of proteins from all five duplicates (**Figure 3**), it is not possible to rule out any VirB6 ortholog as a functional component of the *Rickettsia* T4SS. However, while *virB6a*-*virB6d* genes have arrayed orthologs in *O. tsutsugamushi* and most of the Anaplasmataceae, a *virB6e* ortholog in *O. tsutsugamushi* is not arrayed with *virB6a*-*virB6d*, and no orthologs are present in the Anaplasmataceae genomes (data not shown). This correlates with *virB6e* being the only deleted T4SS scaffold component across 13 *Rickettsia* genomes (*R. massiliae* str. MTU5). Nevertheless, selection for VirB6 duplications in Rickettsiales is evident given the trend for genome reduction [176], and the existence of at least four orthologs hints at a unique and constrained function. VirB6 proteins are similar to ComEC proteins [101], DNA channels involved in the uptake of environmental DNA [177]. Perhaps the *Rickettsia* VirB6 proteins are involved in DNA import, seeding multiple diverse uptake channels to maximize the potential for LGT, particularly in environments (protozoa, macrophage) with a high rate of congener contact. In support of this hypothesis, recent studies identified the *Rickettsia* accessory genome to be predominantly comprised of products and facilitators of the bacterial mobile gene pool [81], [178]. The recent identification of plasmids [79], [179], [180] in *Rickettsia* has shed light on the role of LGT in the sculpting of rickettsial genomes; however, a model for plasmid transfer has not been identified. Our identification of a *Rickettsia* T4SS that is highly similar to the *Legionella lvh* T4SS, which is primarily involved in conjugation [170], suggests a role in conjugation/DNA uptake needs consideration, particularly in light of the persistence of multiple channel proteins that defy the dynamics of rickettsial reductive evolution.

### Lack of *virB5* correlates with rickettsial lifestyle

VirB5 and related proteins (e.g., TraC, TrwJ, LvhB5) are the minor components of the T-pilus [127], [181], [182], [183], as they cofractionate with VirB2 and VirB7 [128], [132] and bind both proteins in yeast two-hybrid screens [134]. *virB6* expression stabilizes cellular levels of VirB5 as well as VirB3 [184], and a VirB5/VirB3 interaction via a yeast two-hybrid screen as well as a pull-down assay further supports the OM localization of VirB5 [134]. Signal sequences are predicted for most of the 157 VirB5 and VirB5-like proteins from diverse bacteria available on GenBank (data not shown). Like VirB2_Ti_, VirB5_Ti_ interacts with VirB1* [119], and immuno-electron microscopy revealed that the protein localizes to the cell-bound region of the pilus as well as the distal regions of the pilus that contact other cells [185]. The crystal structure of TraC (a VirB5 analog) from the IncN plasmid pKM101 revealed a single domain consisting of three bundled helices and a globular appendage [186]. Further site-directed mutagenesis and functional complementation identified 14 highly conserved surface-exposed residues, as well as additional residues involved in TraC dimerization [186]. Despite important knowledge from these studies, the exact role VirB5 and VirB5-like proteins play in T4SS biogenesis and function remains unknown.

blastp searches, as well as other tools designed to detect the strongly conserved residues located at the surface of TraC, failed to identify a VirB5 gene in any of the 13 *Rickettsia* genomes. As evidence is leaning more toward a role of VirB5 in host-cell recognition [185], [186], [187], the lack of a *virB5* in *Rickettsia* is not surprising. A class of surface exposed proteins, Scas, have previously been implicated in host cell recognition and may trigger endocytosis [188]. Also, aside from recent evidence [78], [79], pili have not been observed in *Rickettsia*, and are not evident from all species. The structures reported for *R. felis* [79] and *R. bellii* str. RML 369-C [78] better resemble the flexible and tube-like Gram-negative bacterial conjugative pili, which are quite larger and easier to visualize than the T pilus and related structures [101]. Indeed, a diversity of extracellular pili and pili-like appendages associated with T4SSs exists, including needle like appendages [126], [129], large sheathed structures [189], [190], and fibrous meshes [191]. However, there is little evidence that the T pilus serves as a conduit for substrate transfer [2], [140], and in *A. tumefaciens* T-DNA transfer occurs in the absence of T pilus production [6], [42], [131]. The *B. pertussis* Ptl T4SS also lacks a VirB5 gene, and it has been suggested that such a gene in *B. pertussis* would be redundant, given the absence of cell-cell contact in the secretion of pertussis toxin [24]. Given the strictly intracellular lifestyle of *Rickettsia*, a VirB5 protein (and a T pilus) would also be redundant, as substrates would be secreted and imported directly from the host environment.

### Conclusion

Previous studies on Gram negative bacterial T4SSs have hinted at a reduced scaffold in *Rickettsia* spp. relative to *A. tumefaciens* and other well characterized systems [27] (observations based largely on the *R. prowazekii* genome annotation), and indeed genomic studies on *Rickettsia* as well as other Rickettsiales genera have supported this assessment. However, the composition of the *Rickettsia* T4SS became more complex in recent years, with our past study determining that only four components (VirB1, VirB2, VirB5, VirB7) are missing compared to the *vir* T4SS of *A. tumefaciens* [81]. Reduced T4SSs, wherein only some genes are present within genomes (relative to the *A. tumefaciens vir* archetype), have been suggested to have evolved different functions in certain bacteria [18]. However, trouble lurks within reliance on paradigms (and algorithms). For instance, two T4SSs of *H. pylori*, which independently function in pathogenicity (*cag*) and competence (*comB*), have recently been found to contain more components than automated annotation methods have predicted [164], [165]. Regarding the *cag* system, strong evidence was found for additional putative T4SS components that are not comparable in aa sequence to any of the *vir* proteins [165]. Additionally, diverse components of the divergent *dot/icm* and *lvh* T4SSs of *Legionella* have been demonstrated to complement one another, underscoring the functional resilience of divergent T4SS proteins [27]. Thus caution must be undertaken when depending on automated genome annotation methods for *in silico* characterization of multi-component systems, such as the T4SS. The recent large-scale informatics analysis of 62 bacterial T4SSs revealed a highly conserved core set of T4SS components (VirB6, VirB8-VirB11, VirD4) coupled with a depauperate complex of the remaining Vir components, of which the latter were suggested to have been acquired in various independent events [29]. Our identification of three additional *Rickettsia* T4SS components (VirB1, VirB2, VirB7), all of which comprise much shorter sequences than the more conserved components (**Table 4**), implies that short (under 250 aa) or hyper-variable components are not easily revealed by automated methods. Finally, identifying all components of any secretion system may be hampered when selection does not favor the clustering of all genes within one operon, an apparent characteristic of some obligate intracellular bacteria [150], [192].

**Table 4:** Bioinformatic and Laboratory Evidence for a Functional Rickettsiales *vir* Homolog (*rvh*) T4SS.

Vir^1^	Conservation^2^	Rvh^3^	Size^4^	Evidence^5^
B4	100	B4a	805	A, B, G
		B4b	810	C, D, G
B10	100	B10	483	A, D, G, H
B11	100	B11	334	A, D, E, H
B9	97	B9a	250	E, G
		B9b	158	A, D, Fa, G, H
D4	92	D4	591	D, G, H
B6	90	B6a	426	
		B6b	407	B, G
		B6c	425	Fa, Fb, G
		B6d	420	B, Fa, Fb, G
		B6e	389	Fa, Fb, G
B8	90	B8a	232	C, D
		B8b	243	A, C, D, E, G
B1	53	B1*	252	
B5	52	—	—	—
B2	37	B2*	123	
B3	37	B3	95	
B7	23	B7*	59	C, D, Fa, Fb

1 Vir and Vir-like proteins, following the nomenclature of the *A. tumefaciens vir* system.

2 Percentage of 62 bacterial T4SSs that contain each Vir or Vir-like protein [29].

3 Rickettsiales *vir* homolog. Proteins previously not annotated as Vir via consensus annotation are noted with an asterisk.

4 Average size of the *Rickettsia* orthologs across 13 genome sequences. Numbers for RvhB6a-e show only the VirB6/TrbL domains; avg, lengths of full sized RvhB6 proteins are as follows: RvhB6a = 1055 aa; RvhB6b = 674 aa; RvhB6c = 971 aa; RvhB6d = 886 aa; RvhB6e = 1154 aa.

5 Laboratory studies demonstrating putative function. A = gene expression analysis of *R. conorii* [213]; B = gene expression analysis of *R. prowazekii* [214]; C = gene expression analysis of *R. typhi* [215]; D = bacterial two-hybrid screen of *R. sibirica* [74]; E = proteome analysis of *R. felis* [216]; Fa = PhoA fusion analysis of *R. typhi* [99]; Fb = gene expression analysis of PhoA fusion positive proteins of *R. typhi* [99]; G = proteome analysis of *R. prowazekii* str. Madrd E [217]; H = Differential translation analysis between *R. prowazekii* strains Madrid E and Breinl [218].

Prior seminal reviews of T4SSs have focused largely on pathogenic bacteria, and it has been assumed that the *Rickettsia vir*-like T4SS plays a role in pathogenesis. Our study reveals a highly conserved T4SS across 13 genomes, some of which are not known to be pathogenic to invertebrate and vertebrate hosts. Despite an unknown function of this transporter, laboratory evidence is mounting for at least the expression, regulation and potential secretion of some of its components (**Table 4**). The lack of any identified effector proteins of the *Rickettsia* T4SS, coupled with an as-yet unknown mechanism for the high occurrence of LGT in these genomes, suggests that a function in DNA uptake and translocation, or some modified form of conjugation, cannot be overlooked. It is also possible that the *Rickettsia* T4SS is an amalgam of an effector translocation system and a DNA competence system, especially considering the redundancy of several of its components. These redundant components may serve as environment-specific factors facilitating the persistence of *Rickettsia* in its arthropod and vertebrate hosts, and probable protozoan reservoirs. The affinity to the Legionella *lvh* T4SS, which has recently been shown to serve as a virulence factor in the environmental spreading of Legionnaires' disease [193], suggests the more appropriate annotation of *rvh* (Rickettsiales *vir* homolog) for this evolutionarily intriguing and enigmatic T4SS. Thus, *Legionella* provides a suitable genetic system for future characterization of Rvh scaffold components, as well as for testing the translocation of predicted effector molecules.

## Materials and Methods

### Gene and Protein Sequences

Protocols for manual and automated curation and annotation of predicted rickettsial ORFs are listed at the PATRIC website (http://patric.vbi.vt.edu/about/standard_procedures.php). For the compilation of Rvh components, we merged orthologous groups (OGs) of proteins generated for 12 *Rickettsia* genomes (*R. bellii* str. RML369-C, *R. bellii* str. OSU 85 389, *R. canadensis* str. McKiel, *R. prowazekii* str. Madrid E, *R. typhi* str. Wilmington, *R. felis* str. URRWXCal2, *R. akari* str. Hartford, *R. massilae* str. MTU5, *R. rickettsii* str. Sheila Smith CWPP, *R. conorii* str. Malish 7, *R. sibirica* str. 246, and *R. africae* str. ESF-5) with orthologous sequences from the *R. rickettsii* str. Iowa genome. Consensus annotation identified 15 components (RvhB3, RvhB4a-b RvhB6a-e, RvhB8a-b, RvhB9a-b, RvhB10, RvhB11, RvhD4) and information from the literature (discussed above) guided the identification of three additional components (RvhB1, RvhB2, RvhB7). Gene IDs for all analyzed Vir components across 13 *Rickettsia* genomes are available at the PATRIC website.

### Sequence alignment

All amino acid sequence alignments were performed locally with MUSCLE [194], [195] using default parameters. At least two alignments per Rvh component were performed. First, all 13 *Rickettsia* sequences were aligned (all alignments are shown in **Figure S9**). Second, the *R. typhi* sequences (including duplicate copies if present) were selected for comparison with related proteins from other bacterial T4SSs. Subsets of these latter comparisons (**Figure 2** **–** [Fig pone-0004833-g003] [Fig pone-0004833-g004] [Fig pone-0004833-g005] [Fig pone-0004833-g006] [Fig pone-0004833-g007] **8** **, Figure S2, S3, S5, S6**) differ in their included taxa, reflecting the variability in component presence/absence within bacterial T4SSs, as well as the availability of structural/functional information on particular components. Previously determined structural and/or functional information was superimposed over all alignments (**Figure 2** **–** [Fig pone-0004833-g003] [Fig pone-0004833-g004] [Fig pone-0004833-g005] [Fig pone-0004833-g006] [Fig pone-0004833-g007] **8** **, Figure S2, S3, S5, S6, S9**). The RvhB1 sequences were aligned as follows: the LT domains of *E. coli* soluble lytic transglycosylase Slt70 and the larger rickettsial sequence (RT0388) were aligned using the EMBOSS Needle algorithm (http://ebi.ac.uk/Tools/emboss/align/index.html). This alignment was then fitted to a second alignment based on a needle alignment of *E. coli* Slt70 and the RvhB1 protein from *Brucella suis* [116]. Manual adjustment of the RvhB2 alignment was made based on a prior homology model [122]. The RvhB7 alignment was adjusted around the conserved lipo-processing site and the “P[ILV]NK” motif [141]. The combined alignment of the five RvhB6 proteins was performed using only the VirB6/TrbL domains, which average 413 aa in each duplicate (**Figure S4**). For all 18 *Rickettsia* Rvh components, corresponding nucleotide sequences were aligned with the EMBOSS tranalign tool (http://embossgui.sourceforge.net), using the amino acid alignments as template constraints (**Figure S9**).

### *In silico* characterization

All amino acid sequence comparisons were based on blast results. The nr (All GenBank+RefSeq Nucleotides+EMBL+DDBJ+PDB) database was used, coupled with a search against the Conserved Domains Database. Searches were performed across ‘all organisms’ with composition-based statistics. No filter was used. Default matrix parameters (BLOSUM62) and gap costs (Existence: 11 Extension: 1) were implemented, with an inclusion threshold of 0.005. All Rvh components were screened for possible signal peptides using SignalP [196] and LipoP [197] servers. Sequence logos, when implemented, were created using Weblogo [198], [199]. TMS regions were predicted using the transmembrane hidden Markov model (TMHMM) v.2.0 server [93]. Structural modeling, when implemented, was done using SWISS-MODEL v.8.05 [200].

### Genome structure analysis

Thirteen genome sequences were aligned using Mauve v.2.0.0 [201]. Unmodified Fasta files for each rickettsial genome were used as input, except that the *R. sibirica* genome sequence was re-indexed as before [81] using the reverse-complement of its circular permutation from the original position 668301. Operons were predicted across all thirteen genomes with the program fgenesb [143], using *R. typhi* as the nearest genome and genetic code table 11. The genome browser at PATRIC guided synteny analysis and determination of the genomic positions of the five *rvh* islets.

### Phylogeny estimation

All amino acid sequence alignments were analyzed under parsimony in PAUP* version 4.10 (Altivec) [202]. Heuristic searches implemented 1000 random sequence additions under the tree bisection-reconnection algorithm, with no more than 50 trees saved per replication. Branch support was assessed with 1000 bootstrap replications, except for the combined analysis of all five RvhB6 duplicates (**Figure S4**), in which 500 bootstrap replications were performed, and the combined analysis (**Figure 10**), in which 1 million bootstrap replications were performed. All nucleotide sequence alignments were analyzed under maximum likelihood in PAUP*. Modeltest v3.8 [203] was used to select the best-fit models of evolution for each Rvh gene (**Table S1**). Heuristic searches implemented 500 random sequence additions, saving one tree per replication. Branch support was assessed with 100 bootstrap replications, with 10 random sequence additions per replicate. The combined nucleotide sequences were also analyzed under maximum likelihood using Bayesian inference with the program MrBayes v3.1.2 [204], [205]. Each codon position was modeled separately using the most general time reversible model with gamma distribution and proportion of invariant sites (GTR+G+I). Two independent analyses from different starting seeds were run for three million generations, with samples taken every 1000 generations throughout each analysis using four Markov chains, keeping all chains at the same temperature and saving all branch lengths throughout. Flat priors were implemented. For consensus tree building, burn-in values were determined by plotting log likelihoods and tree lengths over sampled generations in the program Tracer v1.4 [206]. Ultimately, the first 250,000 generations from both analyses were discarded from further evaluation. The estimated sample sizes for all likelihoods, trees and model parameters from both analyses were determined using Tracer to ensure that the MCMC procedure was effectively sampling from the posterior distribution. Tree files from parsimony, maximum likelihood, and Bayesian analyses were used to draw trees in PAUP*.

### Summary statistics

For all alignments, PAUP* was used to determine percent sequence divergence, number of parsimony informative characters, and concordance with the *Rickettsia* species tree. For the nucleotide alignments, average base composition and transition/transversion ratios were also calculated in PAUP*. The ratios of non-synonymous to synonymous substitutions across all nucleotide alignments were calculated using Selecton v2.4 [207].

## Supporting Information

Document S1 Background information describing prior work characterizing the Vir genes previously annotated in Rickettsia genomes.
(0.30 MB DOC)

Figure S1 Illustration of the location of the Vir genes within the R. typhi genome. Note: these 15 Vir genes (green) are a result from consensus annotation of 10 genomes (Gillespie et al. 2008). Two non-Vir genes (yellow) were predicted within vir operons. TU = predicted to be a single transcriptional unit. Operon prediction was performed using fgenesb (Tyson et al. 2004). Note: Region A is within the genome rearrangement unique to R. typhi (McLeod et al. 2004).
(0.58 MB EPS)

Figure S2 Multiple sequence alignment of VirD4 and VirD4-like amino acid sequences across six divergent bacterial species. The secondary structure of TrwB from E. coli plasmid R388 (Gomis-Ruth et al. 2002) is shown above the alignment with arrows (β-strands B2–B11) and bars (α -helices AC-AR). Conserved C-terminal domains 1 and 2 (Schroder et al. 2002) are depicted with black and blue bars, respectively, under the alignment. Conserved motifs I–V within these domains are boxed in red, with motifs I and II enclosing the Walker A (WA) and Walker B (WB) boxes, respectively, involved in ATP binding and hydrolysis. Six structurally proximal residues (including the highly conserved Gln in motif III, denoted with an asterisk) forming the purported NTP-binding cleft (Middleton et al. 2005) are underlined. Coordinates for each sequence are shown to the right in each block, with numbers in parentheses depicting flanking residues of the alignment not shown. Conservation color scheme is the same as in Figure 2 legend. See text for alignment details. Taxon abbreviations and associated NCBI accession numbers are as follows: At = Agrobacterium tumefaciens VirD4, NP_059816; Rt = Rickettsia typhi VirD4, AAU03764; Bh = Bartonella henselae VirD4, CAD89507; Bp = Bordetella pertussis TraG, BAF33479; Lp = Legionella pneumophila LvhD4, CAB60062; Hp = Helicobacter pylori Cag5 (TraG/TraD), NP_207320.
(6.96 MB EPS)

Figure S3 Multiple sequence alignment of VirB11 and VirB11-like amino acid sequences across six divergent bacterial species. A consensus secondary structure from the Helicobacter pylori HP0525 ATPase model (Yeo et al. 2000) and the Brucella suis VirB11 model (Hare et al. 2006) is shown above the alignment with arrows (β-strands B1–B13) and bars (α-helices AA-AJ). Minor discrepancies between both models are stippled over the structural mask. Two helices not predicted in the HP0525 ATPase model are within dashed boxes (α-helices AH2 and AJ). The barrier between the functionally distinct CTD and NTD is shown between β-strand B6 and α-helix AC (Savvides et al. 2003), with associated linker A and B regions of the alignment shaded gray. Residues within linker B that were predicted to form α-helix AC2 (Hare et al. 2006) are colored white. Residues involved in subunit-subunit interactions (Yeo et al. 2000) are depicted with blue circles above the alignment. Residues involved in ADP binding, as well as supposed ATP-binding and hydrolysis, are within black boxes (Rivas et al. 1997; Krause et al. 2000). Colored boxes are as follows: red = Walker A box, green = Asp box, blue = Walker B box, orange = His box (Rivas et al. 1997; Krause et al. 2000). Coordinates for each sequence are shown to the right in each block. Conservation color scheme is the same as in Figure 2 legend. See text for alignment details. Taxon abbreviations and associated NCBI accession numbers are as follows: Hp = H. pylori HP0525, BAD14050; Lp = Legionella pneumophila LvhB11, CAB60061; At = Agrobacterium tumefaciens VirB11, NP_059809; Rt = Rickettsia typhi VirB11, YP_067245; Bh = Bartonella henselae VirB11, YP_034060; Bs = B. suis VirB11, ABY39090.
(5.55 MB EPS)

Figure S4 Phylogeny estimation of five VirB6 proteins and characterization of regions flanking the VirB6/TrbL domain. Phylogeny estimated under parsimony from the amino acid alignment of VirB6/TrbL domains only (see text for details). Branch support is from 500 bootstrap replications. Taxon codes and coloring scheme as described in the Figure 9 legend. Schema at right shows amino acid lengths for VirB6/TrbL domains (red) and flanking additional sequences outside of the VirB6/TrbL domains (black). Flanking regions were Blasted separately from each other and the VirB6/TrbL domains. Each result shows matches to the NCBI conserved domains database and closest non-Rickettsia subject, including its annotation, bit score and E value.
(1.39 MB EPS)

Figure S5 Multiple sequence alignment of VirB10 and VirB10-like amino acid sequences across six divergent bacterial species. The secondary structure of ComB10 from Helicobacter pylori (Terradot et al. 2005) is shown above the alignment with arrows (β-strands B1a-B8b) and bars (α-helices A1–A3). The 310 helix flanking β-strand B1a was not supported by the alignment (dashed box), and bolded residues within this region depict homologous positions under the previous model (Terradot et al. 2005). A Pro-rich tract across the N-terminal region of the alignment is shaded with Pro residues colored white. Blue and black dots depict residues in the crystal packing interface. Coordinates for each sequence are shown to the right in each block. Conservation color scheme is the same as in Figure 2 legend. See text for alignment details. Taxon abbreviations and associated NCBI accession numbers are as follows: Hp = H. pylori ComB, NP_206842-NP_206843; At1 = Agrobacterium tumefaciens TrbI, AAC82638; At2 = A. tumefaciens VirB10, AAK90938; Rt = Rickettsia typhi VirB10, YP_067240; Bs = Brucella suis VirB10, NP_699267; Bh = Bartonella henselae VirB10, CAF28107.
(6.47 MB EPS)

Figure S6 Multiple sequence alignment of VirB3 and VirB3-like amino acid sequences across six divergent bacterial species. Residues near the N-terminus within predicted TMS regions (Krogh et al. 2001) are colored blue. Black bar atop of alignment illustrates portion of alignment wherein all sequences are predicted to be within the TMS region. Black and red boxes enclose previously identified conserved “(GAT)L(ST)RP” and “G” motifs, respectively (Cao and Saier 2001), with an additional invariant Val residue added to the later motif. Shaded N-terminal residues depict signal peptides predicted by SignalP 3.0 (Bendtsen et al. 2004). Coordinates for each sequence are shown to the right. Conservation color scheme is the same as in Figure 2 legend. See text for alignment details. Taxon abbreviations and associated NCBI accession numbers are as follows: At = Agrobacterium tumefaciens VirB3, NP_396489; Rt = Rickettsia typhi VirB3, AAU03522; Bs = Brucella suis VirB3, AAD56613; Bp = Bordetella pertussis PtlB (VirB3), NP_882288; Lp = Legionella pneumophila VirB3, YP_122516; Bh = Bartonella henselae VirB3, AAD48920.
(1.84 MB EPS)

Figure S7 Phylogenetic analysis using Bayesian inference of the combined nucleotide sequences of the Rickettsia T4SS. See text for analysis details. (A) Majority rule consensus of 7400 trees from two independent analyses with removal of burn-in trees. Branch support is from the distribution of posterior probabilities for all branches in the trees. (B) The tree with the best likelihood score (−94170.501) from both independent analysis. (C) Tracer v1.4 plot of the maximum likelihood (LnL) over 3 million generations (sans burn-in) from two independent analyses (black and blue lines), with sampling from every 1,000th generation. (D) Mean and estimated sampling size from the posterior distribution of likelihood, tree and model parameters.
(2.13 MB EPS)

Figure S8 Individual protein and nucleotide phylogeny estimations of 18 Vir components from 13 Rickettsia taxa. Taxon codes and coloring scheme as described in the Figure 9 legend. Protein-based estimations are shown on the left, and were analyzed under parsimony (see text for details). Branch support is from 1,000 bootstrap replications. Inset shows total number of characters (T), number of parsimony informative characters (P), and tree length (L). Single trees are shown as phylograms, whereas consensus cladograms summarize contrasting topologies of all equally parsimonious trees (the number of which are shown in parentheses). Phylogeny estimations from the nucleotide alignments are shown on the right, and were analyzed under maximum likelihood (see text for details). Branch support is from 100 bootstrap replications, with 10 random addition sequences per replicate. Inset shows selected model of evolution as reported by Modeltest, the statistic used to select each model, and the likelihood of the tree.
(0.26 MB PDF)

Figure S9 Amino acid and nucleotide alignments of 18 Vir components across 13 Rickettsia genomes. All alignments are unadjusted from the original MUSCLE alignments performed with default parameters. Structural and functional features for each Vir component (see Figure 2–8, Figure S2, S3, S5, S6) are mapped over the protein alignments. Taxon codes as described in the Figure 9 legend.
(0.51 MB TXT)

Table S1 Best-fit models of evolution via the hierarchical likelihood ratio test (hLRT) and Akaike Information Criterion (AIC) as reported by Modeltest v3.8.
(0.06 MB DOC)
