# Supplementary material for: An Anomalous Type IV Secretion System in Rickettsia Is Evolutionarily Conserved
Source: PLoS One. 2009 Mar 12;4(3):e4833. doi: 10.1371/journal.pone.0004833 (PMC2653234; doi:10.1371/journal.pone.0004833)
Supplement: Document S1 — Background information describing prior work characterizing the Vir genes previously annotated in Rickettsia genomes. (0.30 MB DOC) [file pone.0004833.s001.doc]

**Document S1. Background information describing prior work characterizing the Vir genes that have previously been annotated in *Rickettsia* genomes.**

*Substrate presentation (VirD4)*. VirD4 and related T4SS coupling proteins (T4CPs) form an IM gate that purportedly controls the entry of effector molecules (DNA and/or protein) into the mating channel [1-5]. Substantial insight into the function of these proteins has been gained from the crystal structure of the T4CP TrwB from plasmid R388 of *E*. *coli* [6, 7]. Topologically, T4CPs have two transmembrane-spanning (TMS) regions near the N-terminus that expose a short (~45 aa) sequence to the periplasm, with the majority of the protein remaining cytosolic [8]. The periplasmic N-terminal region has been demonstrated to interact with VirB10 in the mating channel [9-12], and the entire TMS region influences the nucleotide binding properties of the C-terminal domains [13]. The C-terminal portion of the protein is comprised of an IM-proximal α/β nucleotide-binding domain and a cytosolic all-α helical domain [5, 7, 14]. Of five highly conserved motifs within these two domains, two (Walker A and B boxes) contain ATP-binding motifs [15] with demonstrated *in* *vitro* ATP hydrolysis [16, 17]. T4CPs are related to ring-shaped molecular motors, such as sporulation (SpoIIIE) and cell division (FtsK) proteins [18], and form hexameric rings in the IM [6, 7]. Hexamer oligomerization is orchestrated by the less conserved N-terminal sequences [8, 13, 19] but also includes several interactions in the C-terminal cytoplasmic domains [8].

*Translocation energetics (VirB4 and VirB11)*. Like VirD4, VirB4 and related proteins are IM ATPases that form hexameric oligomers [7, 20, 21]. VirB4 is typically the largest component of *vir* systems and contains distinct N- and C-terminal domains (NTD, CTD) with specific functions. The NTD is comprised of the conserved CagE_TrbE_VirB domain (PF03135) that overlaps with a region proposed to mediate VirB4 multimerization [22]. The CTD contains Walker A and B boxes and is predicted to be structurally analogous to TrwB of *Escherichia* *coli* plasmid R388, and hence, VirD4 [23]. Indeed, the Walker A and Walker B motifs and related flanking regions of T4CPs and VirB4/VirB4-like proteins are grouped with other comparable yet diverse proteins, such as bacterial and archaeal repair helicases, bacteriophage ATPases and FtsK ATPases, into the PilT class of NTPases, suggesting an ancient origin of these proteins [24]. While previously considered to be primarily within the IM, three lines of evidence strongly suggest a more periplasmic localization for VirB4 [25]. First, residues have been found to interact between the C-terminus of VirB4 and the N-terminus of VirB11 in yeast two-hybrid screens. Second, *in* *silico* homology modeling supports VirB4 hexamers stacking atop VirB11 hexamers at this defined interaction. Finally, additional yeast two-hybrid interactions occur between the C-terminal region of VirB4 and periplasmic regions of VirB1, VirB8 and VirB10.

VirB11 and VirB11-like proteins, belong to a subclass of PulE NTPases [26], proteins involved in pilus formation and various secretion processes in Gram-negative bacteria [27, 28]. They are essential for type IV secretion [29, 30] and resemble T4CPs and VirB4 and VirB4-like proteins in their IM localization, NTP-binding ability and hexameric structure [20, 31-36]. Specifically, the NTD ring is mostly in contact with the IM and interacts with VirB4 and other Vir channel components [25], and together with the juxtaposed portion of the CTD ring, forms the nucleotide-binding site [20]. Unlike the VirB4 and VirD4 proteins, the CTD of VirB11 is more similar to a RecA domain, with four highly conserved motifs: Walker A box, Asp box, Walker B box, and His Box [31, 35]. The most prominent characteristics of the nucleotide binding site are the lack of conservation of both the Asp residue in the Walker B motif (DhhhhDE, where h = hydrophobic residue) and the C-terminal flanking Glu that are highly conserved in VirB4/VirD4 proteins.

*Mating channel structure* (*VirB6, VirB8-VirB10)*. VirB6 and related proteins are polytopic IM proteins that are essential for type IV secretion [37]. Despite little amino acid conservation across even closely related bacteria [38], elevated hydrophobicity throughout the protein is constrained, supporting its prediction as an IM protein with multiple TMS regions [39, 40]. As a contributor to channel formation, VirB6 and VirB6-like proteins stabilize VirB3 and VirB5, and also facilitate VirB7 dimerization [41] and VirB7-VirB9 heterodimerization [42]. The central and most conserved region of the protein orchestrates the export of DNA through the channel and subsequent transfer to VirB8, while regions within both the C- and N-termini are necessary for substrate transport to VirB2 and VirB9 in the OM [39].

VirB8 and related proteins are bitopic IM channel proteins essential for type IV secretion [37]. The NTD is short and exposed to the cytoplasm, with a single IM-spanning α-helix linking to the larger periplasmic CTD [43-45]. In *A*. *tumefaciens*, VirB8 is the purported scaffold for T4SS polar assembly [40] and several studies have implicated it directly interacting with over half of the Vir components (VirB1, VirB4, VirB5, VirB9-VirB11), as well as itself via dimerization [21, 43, 46-49]. Thus, VirB8 and VirB8-like proteins appear to tether the T pilus complex with the mating channel in T4SSs [21]. Regions of substrate interaction and dimerization occur in the larger CTD [37, 39, 44, 50, 51]. However, an attempt at restoring virulence in a *Bacillus* *suis* VirB8 mutant attenuated system via heterologous complementation with plasmid pSB102 TraJ (a VirB8 homolog) was only successful using a chimera of the *B*. *suis* VirB8 NTD fused to the CTD of TraJ, suggesting a conserved and possibly species specific role for VirB8 NTD [52].

VirB9 and related proteins are secreted to the OM where they likely form the pore of the mating channel [37, 53-55]. The NTD is conserved across diverse bacteria and regulates substrate transfer from the periplasm to the OM [54]. Specifically, interactions between the NTD of VirB9 and VirB10 [43, 54, 56], which are driven by conformational changes in VirB10 upon its sensing of ATP binding by VirB11 and VirD4 [57], are necessary for substrate transfer from the periplasm to the OM [37]. A central region separating the NTD and CTD lacks conservation across diverse bacteria [54] and has been suggested to be extracytoplasmic [58]. The CTD of VirB9 is stabilized by interactions with VirB7 [59-63], and this heterodimer further stabilizes other Vir components in the mating channel [42, 64]. In *A*. *tumefaciens*, a disulphide bond links VirB9_Ti_ and VirB7_Ti_ at residues Cys-262 and Cys-24, respectively [21, 46, 59, 60]; however, the NMR structure of the homologous TraO/TraN complex of the plasmid pKM101 T4SS of *E*. *coli* and bioinformatic analysis of several bacterial VirB9-VirB7 interactions suggest that this disulphide bond is not essential for complex formation [65].

Like VirB8, VirB10 and related proteins are homodimers that span the IM as bitopic proteins with N-terminal proximal TMS regions [66]. However, VirB10 has a larger CTD that bridges the entire periplasm and anchors to the OM via interactions with the VirB9 [43, 54, 56]. Also, the NTDs of VirB10 and VirB10-like proteins interact with T4CPs [9-12], providing a scaffold that links T4CPs to the mating channel and IM Vir components to the OM [1]. VirB8-VirB9-VirB10 complex formation is essential for T4SS function [43, 67], and although VirB10 does not directly contact T-DNA, the effect of its TonB-like energy sensing ability [68] on other Vir components is critical for substrate transfer to the OM channel [37, 57].

*Attachment* (*VirB3)*. VirB3 and related proteins are essential for type IV secretion [1]. Like VirB5, VirB3 and VirB3-like proteins are secreted to the periplasm and OM and are thought to be involved in the formation of the T pilus [69-71]. VirB3 interacts with VirB5 in yeast two hybrid and pull down assays [72] and intracellular levels of both proteins are enhanced by VirB6 expression [41]. VirB4 expression also promotes VirB3 accumulation [73], and an active site VirB4 variant stabilized VirB3 as well as VirB8 [21], suggesting VirB3 interacts with various other Vir components in the periplasm. Despite this, the role VirB3 and VirB3-like proteins play in type IV secretion remains unknown, although it has previously been grouped with VirB1*, VirB2 and VirB5 in the "attachment" category [74].

**References**

**1. Christie PJ: Type IV secretion: the *Agrobacterium* VirB/D4 and related conjugation systems. *Biochim Biophys Acta* 2004, 1694(1-3):219-234.**

**2. Gomis-Ruth FX, de la Cruz F, Coll M: Structure and role of coupling proteins in conjugal DNA transfer. *Res Microbiol* 2002, 153(4):199-204.**

**3. Hamilton CM, Lee H, Li PL, Cook DM, Piper KR, von Bodman SB, Lanka E, Ream W, Farrand SK: TraG from RP4 and TraG and VirD4 from Ti plasmids confer relaxosome specificity to the conjugal transfer system of pTiC58. *J Bacteriol* 2000, 182(6):1541-1548.**

**4. Sastre JI, Cabezon E, de la Cruz F: The carboxyl terminus of protein TraD adds specificity and efficiency to F-plasmid conjugative transfer. *J Bacteriol* 1998, 180(22):6039-6042.**

**5. Schroder G, Krause S, Zechner EL, Traxler B, Yeo HJ, Lurz R, Waksman G, Lanka E: TraG-like proteins of DNA transfer systems and of the *Helicobacter pylori* type IV secretion system: inner membrane gate for exported substrates? *J Bacteriol* 2002, 184(10):2767-2779.**

**6. Gomis-Ruth FX, Coll M: Structure of TrwB, a gatekeeper in bacterial conjugation. *Int J Biochem Cell Biol* 2001, 33(9):839-843.**

**7. Gomis-Ruth FX, Moncalian G, Perez-Luque R, Gonzalez A, Cabezon E, de la Cruz F, Coll M: The bacterial conjugation protein TrwB resembles ring helicases and F1-ATPase. *Nature* 2001, 409(6820):637-641.**

**8. Haft RJ, Gachelet EG, Nguyen T, Toussaint L, Chivian D, Traxler B: *In vivo* oligomerization of the F conjugative coupling protein TraD. *J Bacteriol* 2007, 189(18):6626-6634.**

**9. de Paz HD, Sangari FJ, Bolland S, Garcia-Lobo JM, Dehio C, de la Cruz F, Llosa M: Functional interactions between type IV secretion systems involved in DNA transfer and virulence. *Microbiology* 2005, 151(Pt 11):3505-3516.**

**10. Gilmour MW, Gunton JE, Lawley TD, Taylor DE: Interaction between the IncHI1 plasmid R27 coupling protein and type IV secretion system: TraG associates with the coiled-coil mating pair formation protein TrhB. *Mol Microbiol* 2003, 49(1):105-116.**

**11. Gunton JE, Gilmour MW, Alonso G, Taylor DE: Subcellular localization and functional domains of the coupling protein, TraG, from IncHI1 plasmid R27. *Microbiology* 2005, 151(Pt 11):3549-3561.**

**12. Llosa M, Zunzunegui S, de la Cruz F: Conjugative coupling proteins interact with cognate and heterologous VirB10-like proteins while exhibiting specificity for cognate relaxosomes. *Proc Natl Acad Sci U S A* 2003, 100(18):10465-10470.**

**13. Hormaeche I, Alkorta I, Moro F, Valpuesta JM, Goni FM, De La Cruz F: Purification and properties of TrwB, a hexameric, ATP-binding integral membrane protein essential for R388 plasmid conjugation. *J Biol Chem* 2002, 277(48):46456-46462.**

**14. Gomis-Ruth FX, Moncalian G, de la Cruz F, Coll M: Conjugative plasmid protein TrwB, an integral membrane type IV secretion system coupling protein. Detailed structural features and mapping of the active site cleft. *J Biol Chem* 2002, 277(9):7556-7566.**

**15. Lessl M, Pansegrau W, Lanka E: Relationship of DNA-transfer-systems: essential transfer factors of plasmids RP4, Ti and F share common sequences. *Nucleic Acids Res* 1992, 20(22):6099-6100.**

**16. Tato I, Matilla I, Arechaga I, Zunzunegui S, de la Cruz F, Cabezon E: The ATPase activity of the DNA transporter TrwB is modulated by protein TrwA: implications for a common assembly mechanism of DNA translocating motors. *J Biol Chem* 2007, 282(35):25569-25576.**

**17. Tato I, Zunzunegui S, de la Cruz F, Cabezon E: TrwB, the coupling protein involved in DNA transport during bacterial conjugation, is a DNA-dependent ATPase. *Proc Natl Acad Sci U S A* 2005, 102(23):8156-8161.**

**18. Moncalian G, Cabezon E, Alkorta I, Valle M, Moro F, Valpuesta JM, Goni FM, de La Cruz F: Characterization of ATP and DNA binding activities of TrwB, the coupling protein essential in plasmid R388 conjugation. *J Biol Chem* 1999, 274(51):36117-36124.**

**19. Schroder G, Lanka E: TraG-like proteins of type IV secretion systems: functional dissection of the multiple activities of TraG (RP4) and TrwB (R388). *J Bacteriol* 2003, 185(15):4371-4381.**

**20. Yeo HJ, Savvides SN, Herr AB, Lanka E, Waksman G: Crystal structure of the hexameric traffic ATPase of the *Helicobacter pylori* type IV secretion system. *Mol Cell* 2000, 6(6):1461-1472.**

**21. Yuan Q, Carle A, Gao C, Sivanesan D, Aly KA, Hoppner C, Krall L, Domke N, Baron C: Identification of the VirB4-VirB8-VirB5-VirB2 pilus assembly sequence of type IV secretion systems. *J Biol Chem* 2005, 280(28):26349-26359.**

**22. Dang TA, Zhou XR, Graf B, Christie PJ: Dimerization of the *Agrobacterium tumefaciens* VirB4 ATPase and the effect of ATP-binding cassette mutations on the assembly and function of the T-DNA transporter. *Mol Microbiol* 1999, 32(6):1239-1253.**

**23. Middleton R, Sjolander K, Krishnamurthy N, Foley J, Zambryski P: Predicted hexameric structure of the *Agrobacterium* VirB4 C terminus suggests VirB4 acts as a docking site during type IV secretion. *Proc Natl Acad Sci U S A* 2005, 102(5):1685-1690.**

**24. Constantinesco F, Forterre P, Koonin EV, Aravind L, Elie C: A bipolar DNA helicase gene, *herA*, clusters with *rad50*, *mre11* and *nurA* genes in thermophilic archaea. *Nucleic Acids Res* 2004, 32(4):1439-1447.**

**25. Draper O, Middleton R, Doucleff M, Zambryski PC: Topology of the VirB4 C terminus in the *Agrobacterium tumefaciens* VirB/D4 type IV secretion system. *J Biol Chem* 2006, 281(49):37628-37635.**

**26. Motallebi-Veshareh M, Balzer D, Lanka E, Jagura-Burdzy G, Thomas CM: Conjugative transfer functions of broad-host-range plasmid RK2 are coregulated with vegetative replication. *Mol Microbiol* 1992, 6(7):907-920.**

**27. Hobbs M, Mattick JS: Common components in the assembly of type 4 fimbriae, DNA transfer systems, filamentous phage and protein-secretion apparatus: a general system for the formation of surface-associated protein complexes. *Mol Microbiol* 1993, 10(2):233-243.**

**28. Pugsley AP: The complete general secretory pathway in gram-negative bacteria. *Microbiol Rev* 1993, 57(1):50-108.**

**29. Christie PJ, Vogel JP: Bacterial type IV secretion: conjugation systems adapted to deliver effector molecules to host cells. *Trends Microbiol* 2000, 8(8):354-360.**

**30. Hilleringmann M, Pansegrau W, Doyle M, Kaufman S, MacKichan ML, Gianfaldoni C, Ruggiero P, Covacci A: Inhibitors of *Helicobacter pylori* ATPase Cagalpha block CagA transport and *cag* virulence. *Microbiology* 2006, 152(Pt 10):2919-2930.**

**31. Krause S, Barcena M, Pansegrau W, Lurz R, Carazo JM, Lanka E: Sequence-related protein export NTPases encoded by the conjugative transfer region of RP4 and by the *cag* pathogenicity island of *Helicobacter pylori* share similar hexameric ring structures. *Proc Natl Acad Sci U S A* 2000, 97(7):3067-3072.**

**32. Krause S, Pansegrau W, Lurz R, de la Cruz F, Lanka E: Enzymology of type IV macromolecule secretion systems: the conjugative transfer regions of plasmids RP4 and R388 and the *cag* pathogenicity island of *Helicobacter pylori* encode structurally and functionally related nucleoside triphosphate hydrolases. *J Bacteriol* 2000, 182(10):2761-2770.**

**33. Machon C, Rivas S, Albert A, Goni FM, de la Cruz F: TrwD, the hexameric traffic ATPase encoded by plasmid R388, induces membrane destabilization and hemifusion of lipid vesicles. *J Bacteriol* 2002, 184(6):1661-1668.**

**34. Rashkova S, Spudich GM, Christie PJ: Characterization of membrane and protein interaction determinants of the *Agrobacterium tumefaciens* VirB11 ATPase. *J Bacteriol* 1997, 179(3):583-591.**

**35. Rivas S, Bolland S, Cabezon E, Goni FM, de la Cruz F: TrwD, a protein encoded by the IncW plasmid R388, displays an ATP hydrolase activity essential for bacterial conjugation. *J Biol Chem* 1997, 272(41):25583-25590.**

**36. Savvides SN, Yeo HJ, Beck MR, Blaesing F, Lurz R, Lanka E, Buhrdorf R, Fischer W, Haas R, Waksman G: VirB11 ATPases are dynamic hexameric assemblies: new insights into bacterial type IV secretion. *Embo J* 2003, 22(9):1969-1980.**

**37. Cascales E, Christie PJ: Definition of a bacterial type IV secretion pathway for a DNA substrate. *Science* 2004, 304(5674):1170-1173.**

**38. Judd PK, Mahli D, Das A: Molecular characterization of the *Agrobacterium tumefaciens* DNA transfer protein VirB6. *Microbiology* 2005, 151(Pt 11):3483-3492.**

**39. Jakubowski SJ, Krishnamoorthy V, Cascales E, Christie PJ: *Agrobacterium tumefaciens* VirB6 domains direct the ordered export of a DNA substrate through a type IV secretion system. *J Mol Biol* 2004, 341(4):961-977.**

**40. Judd PK, Kumar RB, Das A: The type IV secretion apparatus protein VirB6 of *Agrobacterium tumefaciens* localizes to a cell pole. *Mol Microbiol* 2005, 55(1):115-124.**

**41. Hapfelmeier S, Domke N, Zambryski PC, Baron C: VirB6 is required for stabilization of VirB5 and VirB3 and formation of VirB7 homodimers in *Agrobacterium tumefaciens*. *J Bacteriol* 2000, 182(16):4505-4511.**

**42. Jakubowski SJ, Krishnamoorthy V, Christie PJ: *Agrobacterium tumefaciens* VirB6 protein participates in formation of VirB7 and VirB9 complexes required for type IV secretion. *J Bacteriol* 2003, 185(9):2867-2878.**

**43. Das A, Xie YH: The *Agrobacterium* T-DNA transport pore proteins VirB8, VirB9, and VirB10 interact with one another. *J Bacteriol* 2000, 182(3):758-763.**

**44. Terradot L, Bayliss R, Oomen C, Leonard GA, Baron C, Waksman G: Structures of two core subunits of the bacterial type IV secretion system, VirB8 from *Brucella suis* and ComB10 from *Helicobacter pylori*. *Proc Natl Acad Sci U S A* 2005, 102(12):4596-4601.**

**45. Thorstenson YR, Zambryski PC: The essential virulence protein VirB8 localizes to the inner membrane of *Agrobacterium tumefaciens*. *J Bacteriol* 1994, 176(6):1711-1717.**

**46. Hoppner C, Carle A, Sivanesan D, Hoeppner S, Baron C: The putative lytic transglycosylase VirB1 from *Brucella suis* interacts with the type IV secretion system core components VirB8, VirB9 and VirB11. *Microbiology* 2005, 151(Pt 11):3469-3482.**

**47. Judd PK, Kumar RB, Das A: Spatial location and requirements for the assembly of the *Agrobacterium tumefaciens* type IV secretion apparatus. *Proc Natl Acad Sci U S A* 2005, 102(32):11498-11503.**

**48. Kumar RB, Xie YH, Das A: Subcellular localization of the *Agrobacterium tumefaciens* T-DNA transport pore proteins: VirB8 is essential for the assembly of the transport pore. *Mol Microbiol* 2000, 36(3):608-617.**

**49. Ward DV, Draper O, Zupan JR, Zambryski PC: Peptide linkage mapping of the *Agrobacterium tumefaciens vir*-encoded type IV secretion system reveals protein subassemblies. *Proc Natl Acad Sci U S A* 2002, 99(17):11493-11500.**

**50. Bailey S, Ward D, Middleton R, Grossmann JG, Zambryski PC: *Agrobacterium tumefaciens* VirB8 structure reveals potential protein-protein interaction sites. *Proc Natl Acad Sci U S A* 2006, 103(8):2582-2587.**

**51. Paschos A, Patey G, Sivanesan D, Gao C, Bayliss R, Waksman G, O'Callaghan D, Baron C: Dimerization and interactions of *Brucella suis* VirB8 with VirB4 and VirB10 are required for its biological activity. *Proc Natl Acad Sci U S A* 2006, 103(19):7252-7257.**

**52. Patey G, Qi Z, Bourg G, Baron C, O'Callaghan D: Swapping of periplasmic domains between *Brucella suis* VirB8 and a pSB102 VirB8 homologue allows heterologous complementation. *Infect Immun* 2006, 74(8):4945-4949.**

**53. Atmakuri K, Cascales E, Christie PJ: Energetic components VirD4, VirB11 and VirB4 mediate early DNA transfer reactions required for bacterial type IV secretion. *Mol Microbiol* 2004, 54(5):1199-1211.**

**54. Jakubowski SJ, Cascales E, Krishnamoorthy V, Christie PJ: *Agrobacterium tumefaciens* VirB9, an outer-membrane-associated component of a type IV secretion system, regulates substrate selection and T-pilus biogenesis. *J Bacteriol* 2005, 187(10):3486-3495.**

**55. Lawley TD, Klimke WA, Gubbins MJ, Frost LS: F factor conjugation is a true type IV secretion system. *FEMS Microbiol Lett* 2003, 224(1):1-15.**

**56. Beaupre CE, Bohne J, Dale EM, Binns AN: Interactions between VirB9 and VirB10 membrane proteins involved in movement of DNA from *Agrobacterium tumefaciens* into plant cells. *J Bacteriol* 1997, 179(1):78-89.**

**57. Cascales E, Christie PJ: *Agrobacterium* VirB10, an ATP energy sensor required for type IV secretion. *Proc Natl Acad Sci U S A* 2004, 101(49):17228-17233.**

**58. Cao TB, Saier MH, Jr.: Conjugal type IV macromolecular transfer systems of Gram-negative bacteria: organismal distribution, structural constraints and evolutionary conclusions. *Microbiology* 2001, 147(Pt 12):3201-3214.**

**59. Anderson LB, Hertzel AV, Das A: *Agrobacterium tumefaciens* VirB7 and VirB9 form a disulfide-linked protein complex. *Proc Natl Acad Sci U S A* 1996, 93(17):8889-8894.**

**60. Baron C, Thorstenson YR, Zambryski PC: The lipoprotein VirB7 interacts with VirB9 in the membranes of *Agrobacterium tumefaciens*. *J Bacteriol* 1997, 179(4):1211-1218.**

**61. Bayan N, Guilvout I, Pugsley AP: Secretins take shape. *Mol Microbiol* 2006, 60(1):1-4.**

**62. Fernandez D, Dang TA, Spudich GM, Zhou XR, Berger BR, Christie PJ: The *Agrobacterium tumefaciens* *virB7* gene product, a proposed component of the T-complex transport apparatus, is a membrane-associated lipoprotein exposed at the periplasmic surface. *J Bacteriol* 1996, 178(11):3156-3167.**

**63. Spudich GM, Fernandez D, Zhou XR, Christie PJ: Intermolecular disulfide bonds stabilize VirB7 homodimers and VirB7/VirB9 heterodimers during biogenesis of the *Agrobacterium tumefaciens* T-complex transport apparatus. *Proc Natl Acad Sci U S A* 1996, 93(15):7512-7517.**

**64. Fernandez D, Spudich GM, Zhou XR, Christie PJ: The *Agrobacterium tumefaciens* VirB7 lipoprotein is required for stabilization of VirB proteins during assembly of the T-complex transport apparatus. *J Bacteriol* 1996, 178(11):3168-3176.**

**65. Bayliss R, Harris R, Coutte L, Monier A, Fronzes R, Christie PJ, Driscoll PC, Waksman G: NMR structure of a complex between the VirB9/VirB7 interaction domains of the pKM101 type IV secretion system. *Proc Natl Acad Sci U S A* 2007, 104(5):1673-1678.**

**66. Das A, Xie YH: Construction of transposon Tn*3phoA*: its application in defining the membrane topology of the *Agrobacterium tumefaciens* DNA transfer proteins. *Mol Microbiol* 1998, 27(2):405-414.**

**67. Kumar RB, Das A: Functional analysis of the *Agrobacterium tumefaciens* T-DNA transport pore protein VirB8. *J Bacteriol* 2001, 183(12):3636-3641.**

**68. Postle K, Kadner RJ: Touch and go: tying TonB to transport. *Mol Microbiol* 2003, 49(4):869-882.**

**69. Krall L, Wiedemann U, Unsin G, Weiss S, Domke N, Baron C: Detergent extraction identifies different VirB protein subassemblies of the type IV secretion machinery in the membranes of *Agrobacterium tumefaciens*. *Proc Natl Acad Sci U S A* 2002, 99(17):11405-11410.**

**70. Lai EM, Eisenbrandt R, Kalkum M, Lanka E, Kado CI: Biogenesis of T pili in *Agrobacterium tumefaciens* requires precise VirB2 propilin cleavage and cyclization. *J Bacteriol* 2002, 184(1):327-330.**

**71. Schmidt-Eisenlohr H, Domke N, Angerer C, Wanner G, Zambryski PC, Baron C: Vir proteins stabilize VirB5 and mediate its association with the T pilus of *Agrobacterium tumefaciens*. *J Bacteriol* 1999, 181(24):7485-7492.**

**72. Shamaei-Tousi A, Cahill R, Frankel G: Interaction between protein subunits of the type IV secretion system of *Bartonella henselae*. *J Bacteriol* 2004, 186(14):4796-4801.**

**73. Jones AL, Shirasu K, Kado CI: The product of the *virB4* gene of *Agrobacterium tumefaciens* promotes accumulation of VirB3 protein. *J Bacteriol* 1994, 176(17):5255-5261.**

**74. Christie PJ: Type IV secretion: intercellular transfer of macromolecules by systems ancestrally related to conjugation machines. *Mol Microbiol* 2001, 40(2):294-305.**
