# Supplementary material for: An Anomalous Type IV Secretion System in Rickettsia Is Evolutionarily Conserved
Source: PLoS One. 2009 Mar 12;4(3):e4833. doi: 10.1371/journal.pone.0004833 (PMC2653234; doi:10.1371/journal.pone.0004833)
Supplement: Figure S8 — Individual protein and nucleotide phylogeny estimations of 18 Vir components from 13 Rickettsia taxa. Taxon codes and coloring scheme as described in the Figure 9 legend. Protein-based estimations are shown on the left, and were analyzed under parsimony (see text for details). Branch support is from 1,000 bootstrap replications. Inset shows total number of characters (T), number of parsimony informative characters (P), and tree length (L). Single trees are shown as phylograms, whereas consensus cladograms summarize contrasting topologies of all equally parsimonious trees (the number of which are shown in parentheses). Phylogeny estimations from the nucleotide alignments are shown on the right, and were analyzed under maximum likelihood (see text for details). Branch support is from 100 bootstrap replications, with 10 random addition sequences per replicate. Inset shows selected model of evolution as reported by Modeltest, the statistic used to select each model, and the likelihood of the tree. (0.26 MB PDF) [file pone.0004833.s009.pdf]

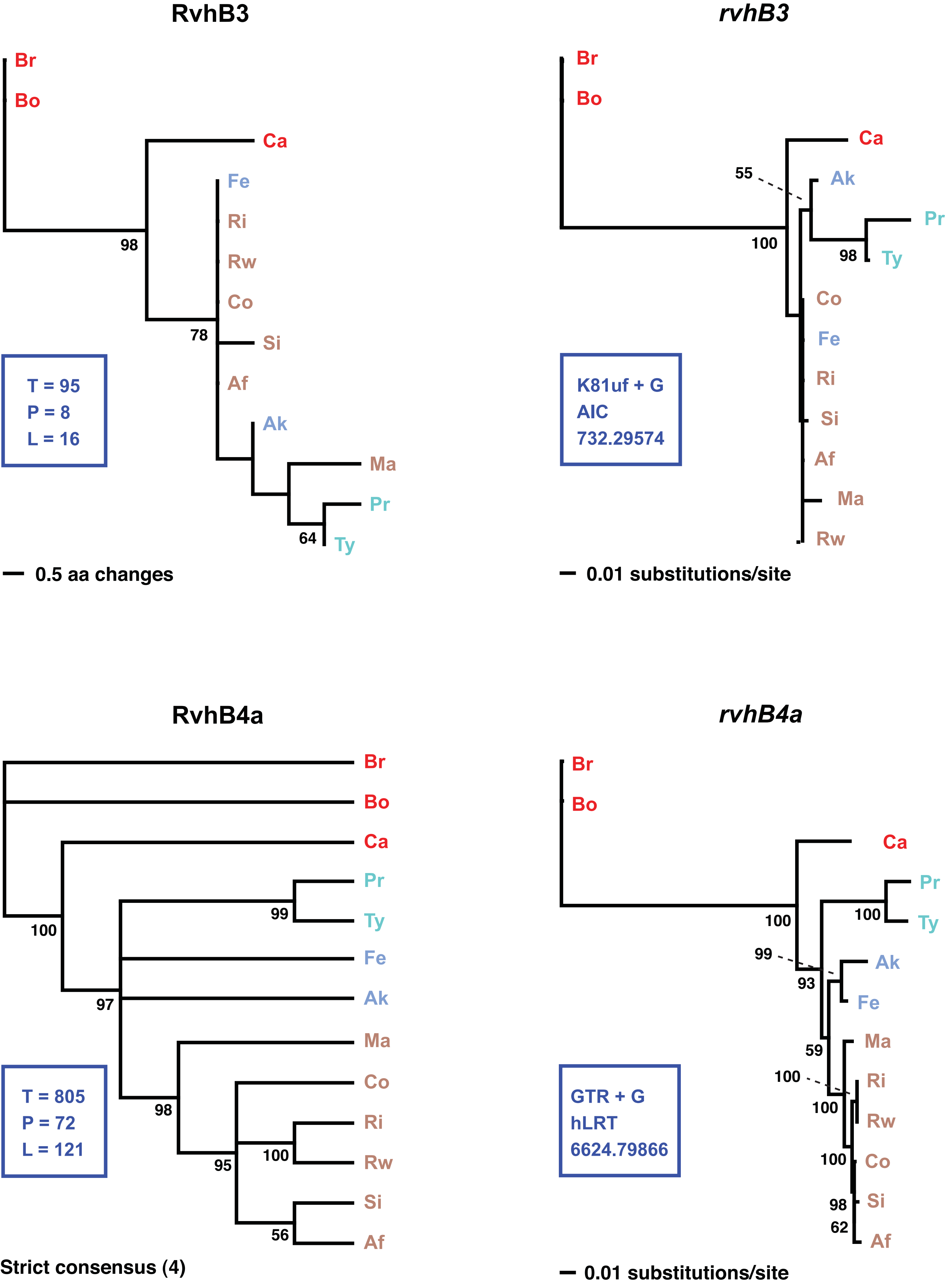
RvhB3
Br
Bo
Ca
Fe
Ri
Rw
Co
Si
Af
Ak
Ma
Pr
Ty
98
78
64
T = 95
P = 8
L = 16
0.5 aa changes
rvhB3
Br
Bo
Ca
55
Ak
Pr
100
98
Ty
Co
Fe
Ri
Si
Af
Ma
Rw
K81uf + G
AIC
732.29574
0.01 substitutions/site
RvhB4a
Br
Bo
Ca
Pr
Ty
Fe
Ak
Ma
Co
Ri
Rw
Si
Af
100
99
97
98
95
100
56
T = 805
P = 72
L = 121
Strict consensus (4)
rvhB4a
Br
Bo
Ca
Pr
Ty
Ak
Fe
Ma
Ri
Rw
Co
Si
Af
100
100
99
93
59
100
100
100
98
62
GTR + G
hLRT
6624.79866
0.01 substitutions/site

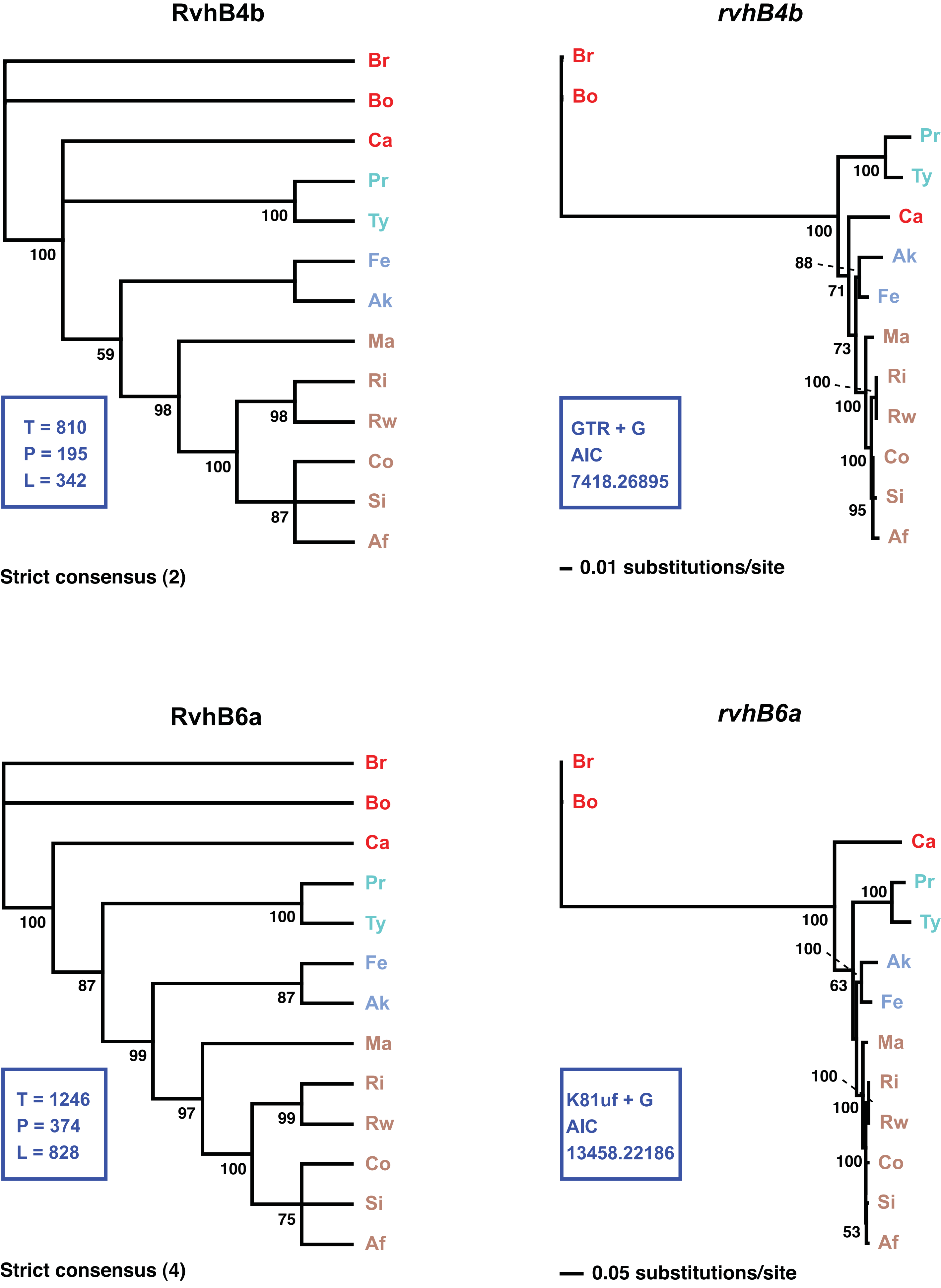
RvhB4b
Br
Bo
Ca
Pr
Ty
Fe
Ak
Ma
Ri
Rw
Co
Si
Af
100
100
59
98
98
100
87
T = 810
P = 195
L = 342
Strict consensus (2)
rvhB4b
Br
Bo
Pr
Ty
Ca
Ak
Fe
Ma
Ri
Rw
Co
Si
Af
100
100
88
71
73
100
100
100
95
GTR + G
AIC
7418.26895
0.01 substitutions/site
RvhB6a
Br
Bo
Ca
Pr
Ty
Fe
Ak
Ma
Ri
Rw
Co
Si
Af
100
100
87
87
99
97
99
100
75
T = 1246
P = 374
L = 828
Strict consensus (4)
rvhB6a
Br
Bo
Ca
Pr
Ty
Ak
Fe
Ma
Ri
Rw
Co
Si
Af
100
100
100
63
100
100
100
53
K81uf + G
AIC
13458.22186
0.05 substitutions/site

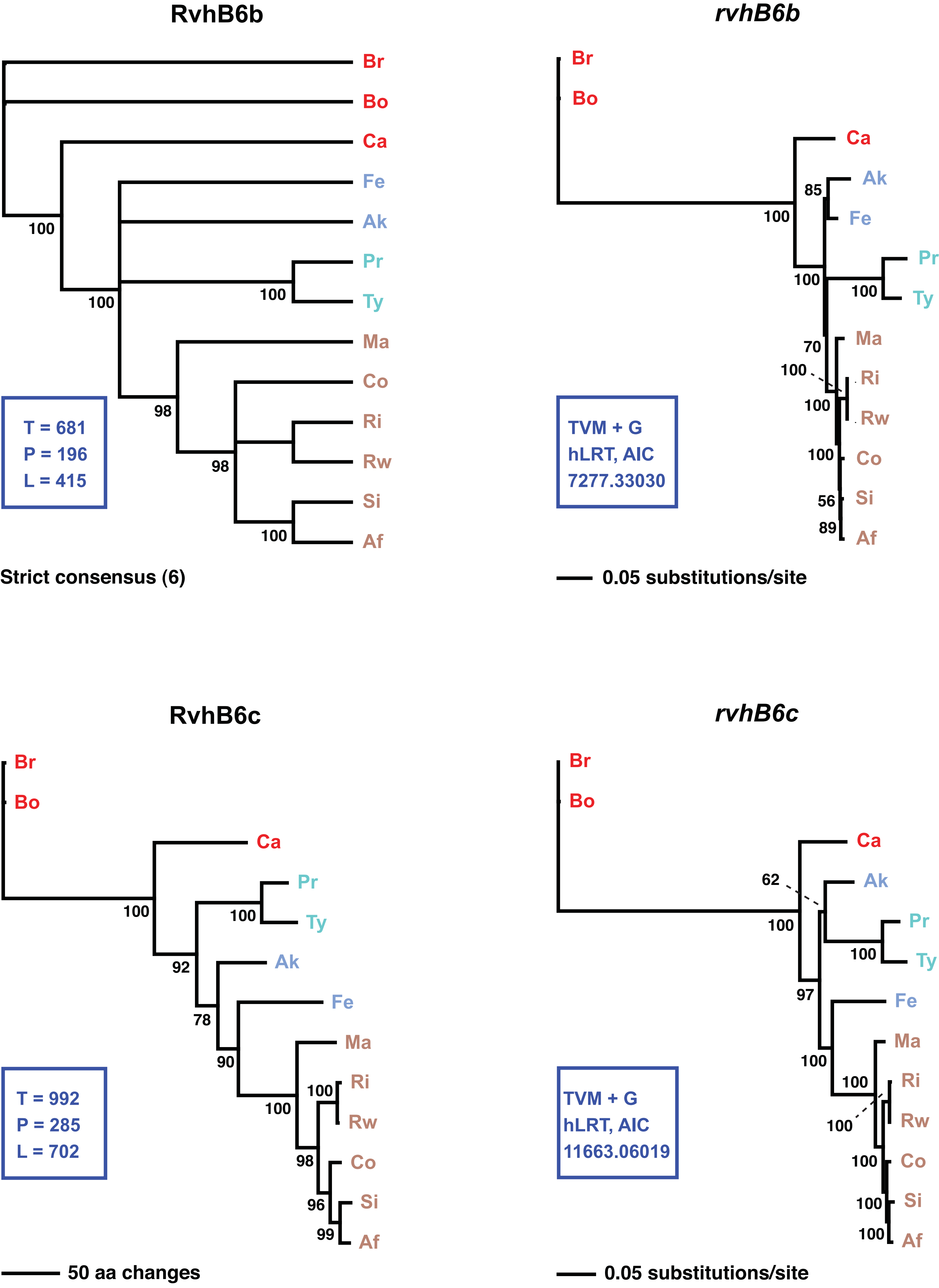

RvhB6b
Br
Bo
Ca
Fe
Ak
Pr
Ty
Ma
Co
Ri
Rw
Si
Af
100
100
100
98
98
100
T = 681
P = 196
L = 415
Strict consensus (6)
rvhB6b
Br
Bo
Ca
Ak
Fe
Pr
Ty
Ma
Ri
Rw
Co
Si
Af
85
100
100
100
70
100
100
100
56
89
TVM + G
hLRT, AIC
7277.33030
0.05 substitutions/site
RvhB6c
Br
Bo
Ca
Pr
Ty
Ak
Fe
Ma
Ri
Rw
Co
Si
Af
100
100
92
78
90
100
100
98
96
99
T = 992
P = 285
L = 702
50 aa changes
rvhB6c
Br
Bo
Ca
Ak
Pr
Ty
Fe
Ma
Ri
Rw
Co
Si
Af
62
100
100
97
100
100
100
100
100
100
TVM + G
hLRT, AIC
11663.06019
0.05 substitutions/site

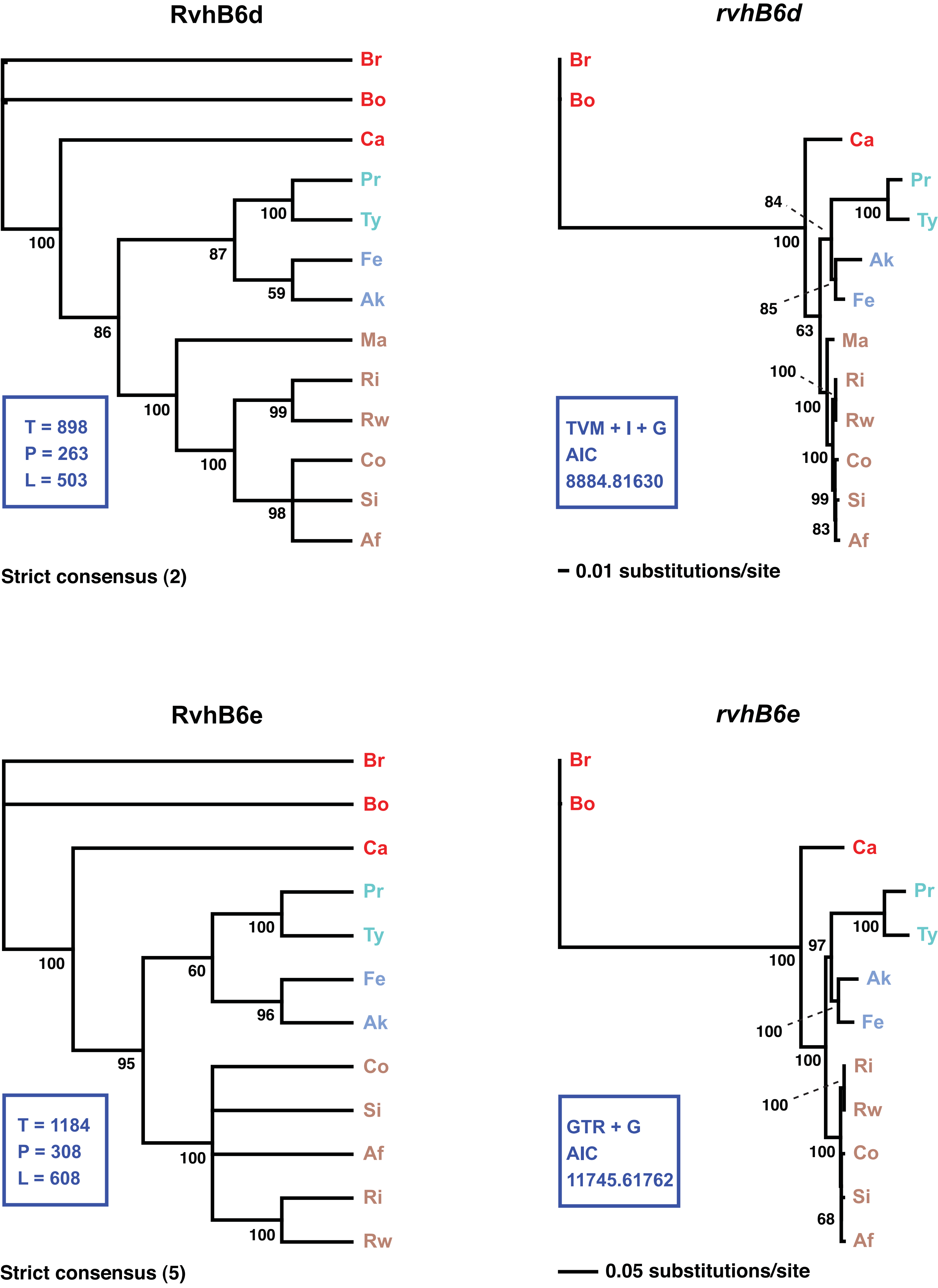
RvhB6d
Br
Bo
Ca
Pr
Ty
Fe
Ak
Ma
Ri
Rw
Co
Si
Af
100
100
87
59
86
100
99
100
98
T = 898
P = 263
L = 503
Strict consensus (2)
rvhB6d
Br
Bo
Ca
Pr
Ty
Ak
Fe
Ma
Ri
Rw
Co
Si
Af
84
100
100
85
63
100
100
100
99
83
TVM + I + G
AIC
8884.81630
0.01 substitutions/site
RvhB6e
Br
Bo
Ca
Pr
Ty
Fe
Ak
Co
Si
Af
Ri
Rw
100
100
60
96
95
100
100
T = 1184
P = 308
L = 608
Strict consensus (5)
rvhB6e
Br
Bo
Ca
Pr
Ty
Ak
Fe
Ri
Rw
Co
Si
Af
100
97
100
100
100
100
100
68
GTR + G
AIC
11745.61762
0.05 substitutions/site

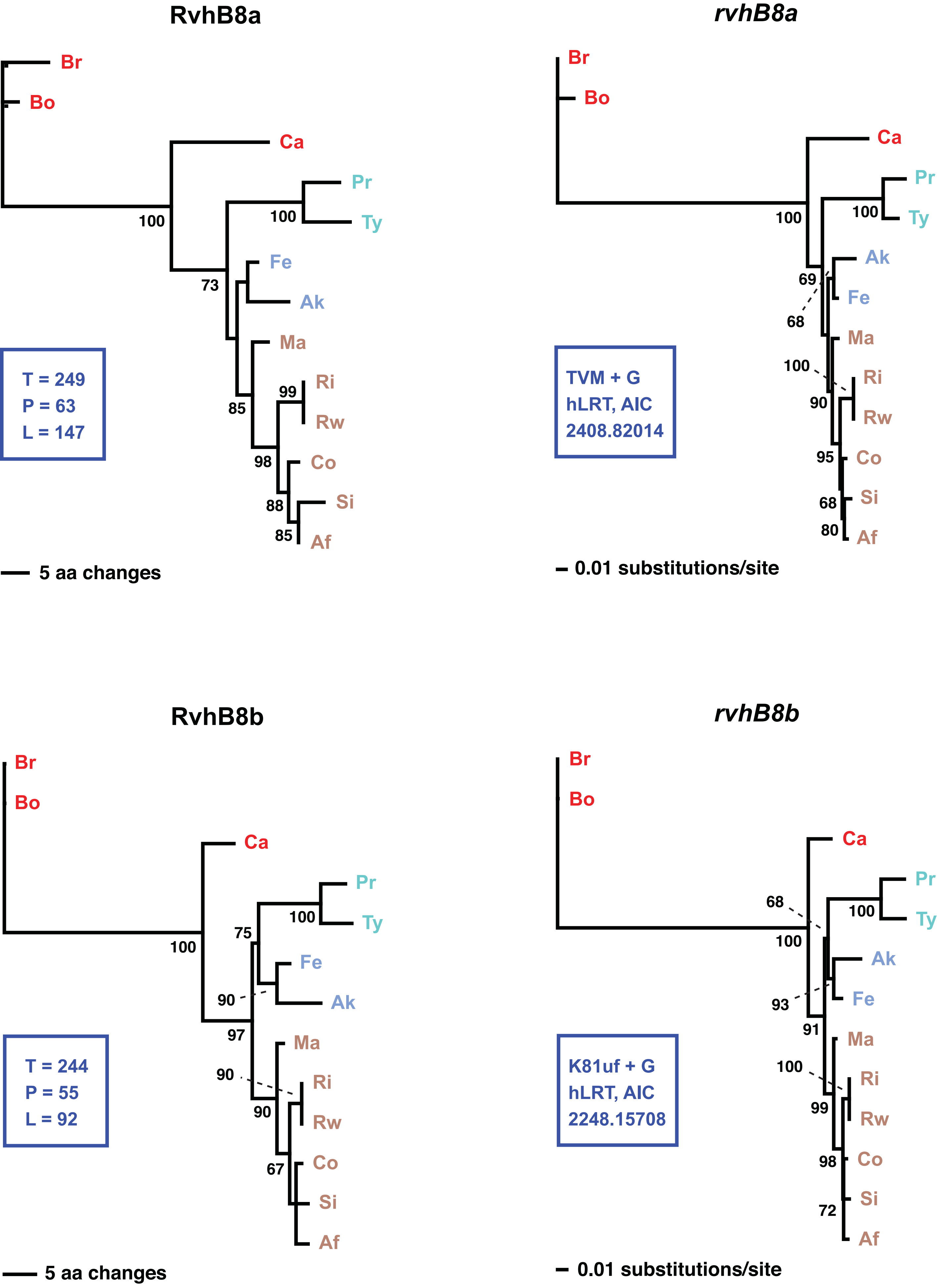
RvhB8a
Br
Bo
Ca
Pr
Ty
Fe
Ak
Ma
Ri
Rw
Co
Si
Af
100
100
73
85
99
98
88
85
T = 249
P = 63
L = 147
5 aa changes
rvhB8a
Br
Bo
Ca
Pr
Ty
Ak
Fe
Ma
Ri
Rw
Co
Si
Af
100
100
69
68
100
90
95
68
80
TVM + G
hLRT, AIC
2408.82014
0.01 substitutions/site
RvhB8b
Br
Bo
Ca
Pr
Ty
Fe
Ak
Ma
Ri
Rw
Co
Si
Af
100
100
75
90
97
90
90
67
T = 244
P = 55
L = 92
5 aa changes
rvhB8b
Br
Bo
Ca
Pr
Ty
Ak
Fe
Ma
Ri
Rw
Co
Si
Af
68
100
100
93
91
100
99
98
72
K81uf + G
hLRT, AIC
2248.15708
0.01 substitutions/site

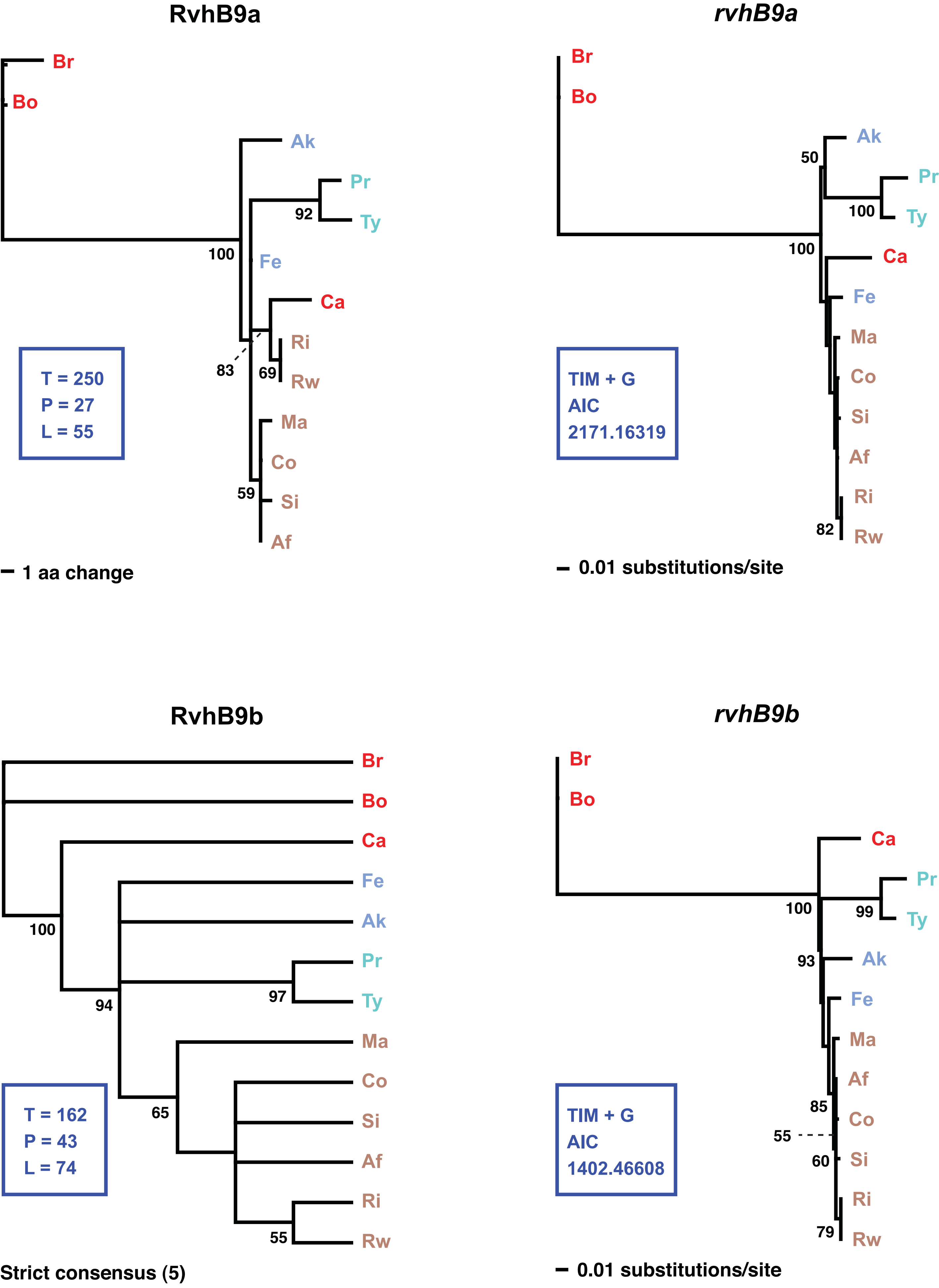
RvhB9a
Br
Bo
Ak
Pr
Ty
92
100
Fe
Ca
Ri
83
69
Rw
Ma
Co
59
Si
Af
T = 250
P = 27
L = 55
1 aa change
rvhB9a
Br
Bo
Ak
50
Pr
100
Ty
100
Ca
Fe
Ma
Co
Si
Af
Ri
82
Rw
TIM + G
AIC
2171.16319
0.01 substitutions/site
RvhB9b
Br
Bo
Ca
Fe
Ak
100
Pr
97
Ty
94
Ma
Co
65
Si
Af
Ri
55
Rw
T = 162
P = 43
L = 74
Strict consensus (5)
rvhB9b
Br
Bo
Ca
Pr
100
99
Ty
93
Ak
Fe
Ma
Af
85
Co
55
60
Si
Ri
79
Rw
TIM + G
AIC
1402.46608
0.01 substitutions/site

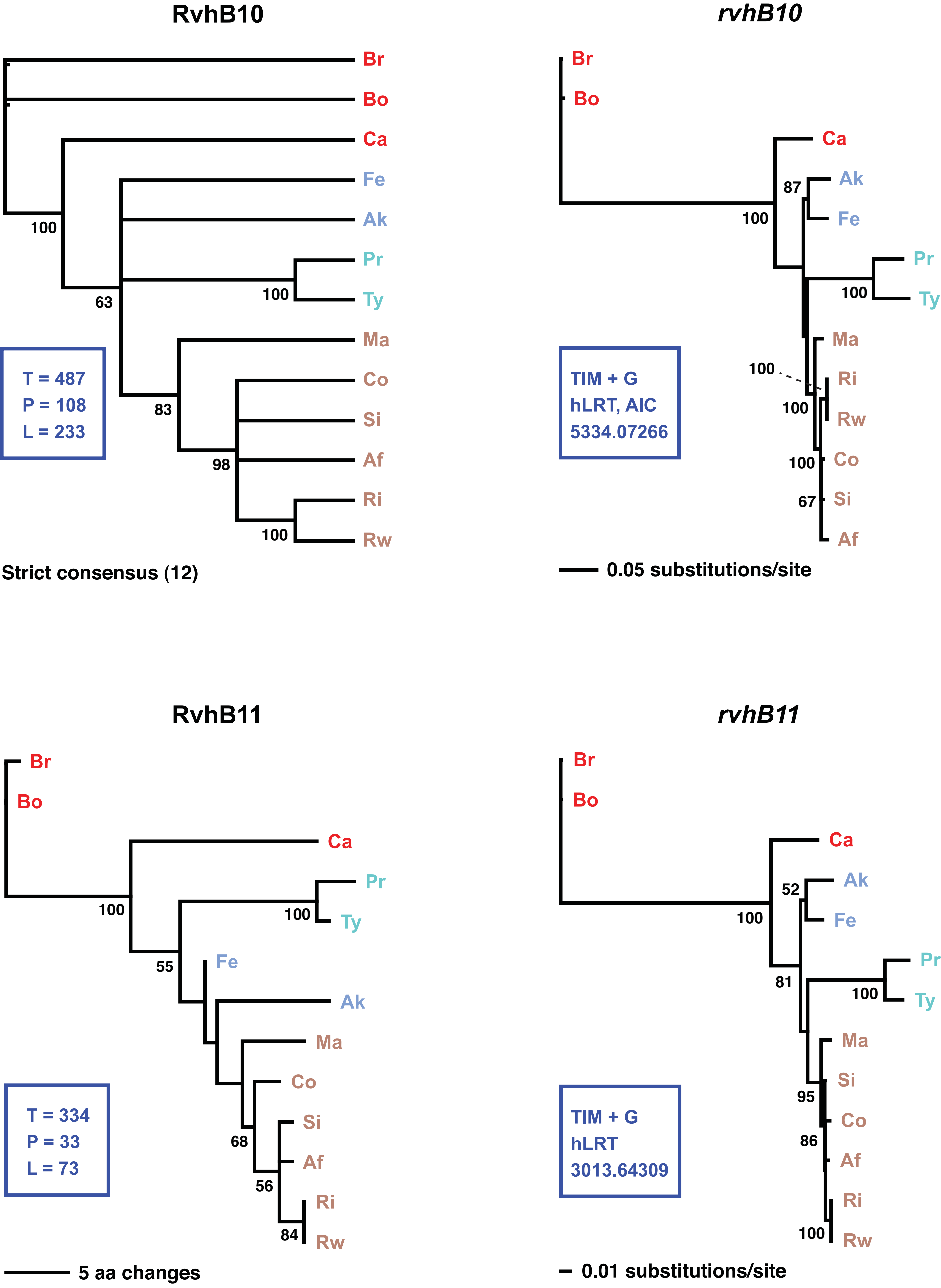
RvhB10
Br
Bo
Ca
Fe
Ak
Pr
Ty
Ma
Co
Si
Af
Ri
Rw
100
63
100
83
98
100
T = 487
P = 108
L = 233
Strict consensus (12)
rvhB10
Br
Bo
Ca
Ak
Fe
Pr
Ty
Ma
Ri
Rw
Co
Si
Af
87
100
100
100
100
100
67
TIM + G
hLRT, AIC
5334.07266
0.05 substitutions/site
RvhB11
Br
Bo
Ca
Pr
Ty
Fe
Ak
Ma
Co
Si
Af
Ri
Rw
100
100
55
68
56
84
T = 334
P = 33
L = 73
5 aa changes
rvhB11
Br
Bo
Ca
Ak
Fe
Pr
Ty
Ma
Si
Co
Af
Ri
Rw
52
100
81
100
95
86
100
TIM + G
hLRT
3013.64309
0.01 substitutions/site

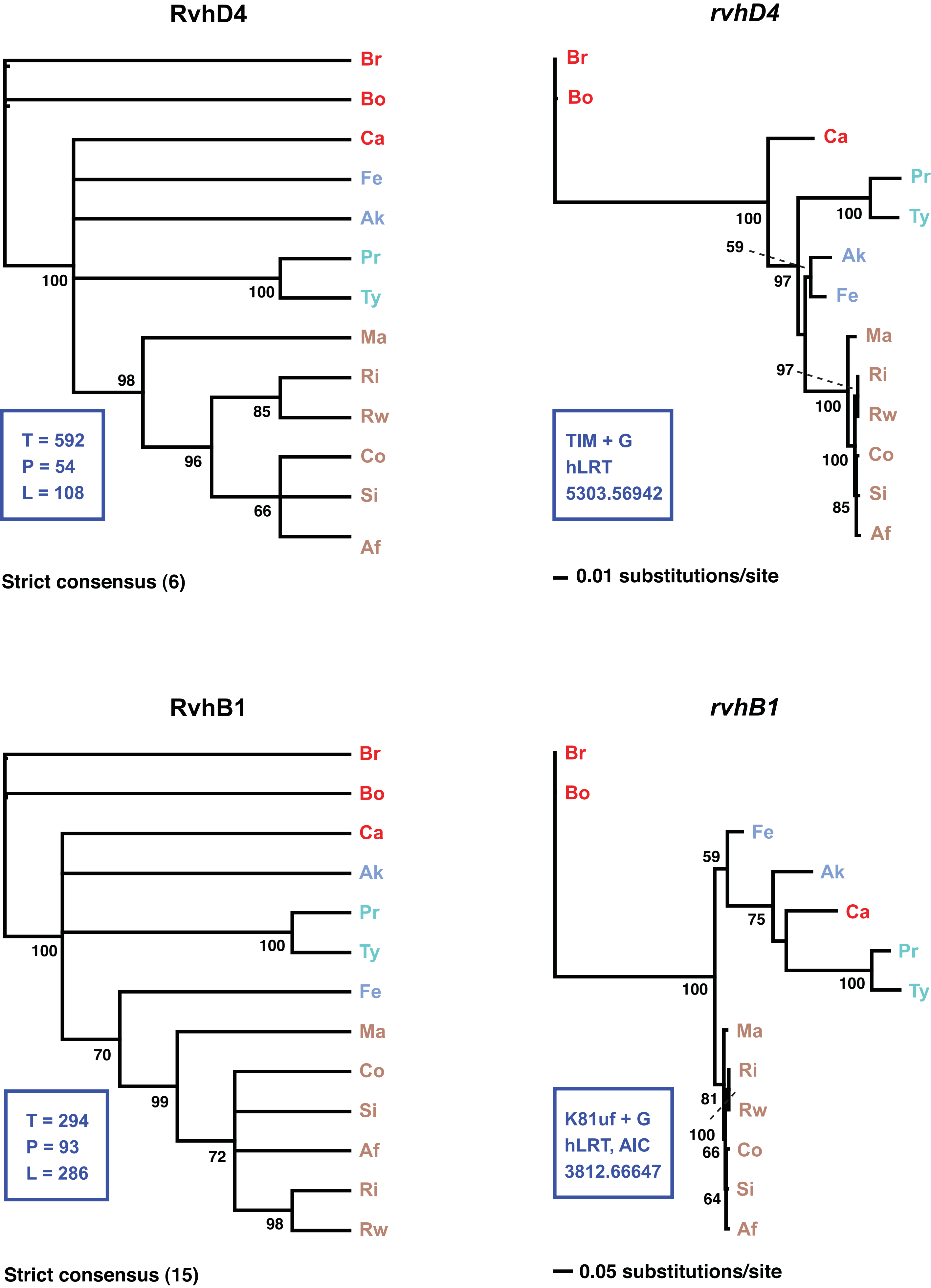

RvhD4
Br
Bo
Ca
Fe
Ak
Pr
Ty
Ma
Ri
Rw
Co
Si
Af
100
100
98
85
96
66
T = 592
P = 54
L = 108
Strict consensus (6)
rvhD4
Br
Bo
Ca
Pr
Ty
Ak
Fe
Ma
Ri
Rw
Co
Si
Af
100
100
59
97
97
100
100
85
TIM + G
hLRT
5303.56942
0.01 substitutions/site
RvhB1
Br
Bo
Ca
Ak
Pr
Ty
Fe
Ma
Co
Si
Af
Ri
Rw
100
100
70
99
72
98
T = 294
P = 93
L = 286
Strict consensus (15)
rvhB1
Br
Bo
Fe
Ak
Ca
Pr
Ty
Ma
Ri
Rw
Co
Si
Af
59
75
100
100
81
100
66
64
K81uf + G
hLRT, AIC
3812.66647
0.05 substitutions/site

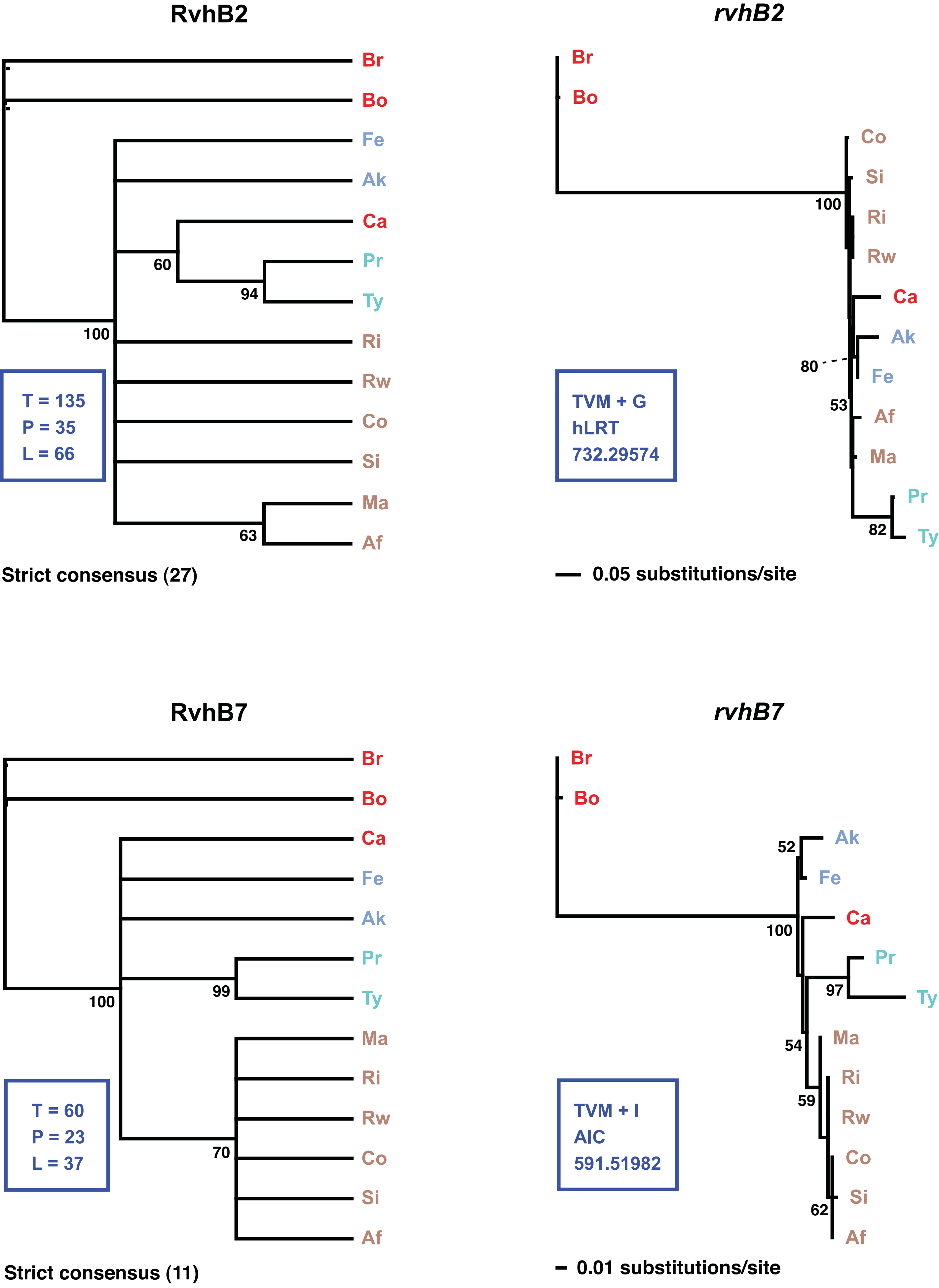
RvhB2
Br
Bo
Fe
Ak
Ca
Pr
Ty
Ri
Rw
Co
Si
Ma
Af
60
94
100
63
T = 135
P = 35
L = 66
Strict consensus (27)
rvhB2
Br
Bo
Co
Si
Ri
Rw
Ca
Ak
Fe
Af
Ma
Pr
Ty
100
80
53
82
TVM + G
hLRT
732.29574
0.05 substitutions/site
RvhB7
Br
Bo
Ca
Fe
Ak
Pr
Ty
Ma
Ri
Rw
Co
Si
Af
99
100
70
T = 60
P = 23
L = 37
Strict consensus (11)
rvhB7
Br
Bo
Ak
Fe
Ca
Pr
Ty
Ma
Ri
Rw
Co
Si
Af
52
100
97
54
59
62
TVM + I
AIC
591.51982
0.01 substitutions/site
