# Supplementary material for: An Anomalous Type IV Secretion System in Rickettsia Is Evolutionarily Conserved
Source: PLoS One. 2009 Mar 12;4(3):e4833. doi: 10.1371/journal.pone.0004833 (PMC2653234; doi:10.1371/journal.pone.0004833)
Supplement: Table S1 — Best-fit models of evolution via the hierarchical likelihood ratio test (hLRT) and Akaike Information Criterion (AIC) as reported by Modeltest v3.8. (0.06 MB DOC) [file pone.0004833.s011.doc]

**Table S1. Best-fit models of evolution via the hierarchical likelihood ratio test (hLRT) and Akaike Information Criterion (AIC) as reported by Modeltest v3.8.**

**Gene^1^ hLRT** **AIC**

**Model^2^ - lnL K α Model^2^ -lnL K α**

*virB3* HKY+G 737.2280 28 0.3245 **K81uf+G** 734.5382 29 0.2874

*virB4a* **GTR+G** 6631.7402 32 0.1305 TIM+I+G 6631.8066 31 2.1245

*virB4b* TVM+G 7338.3901 31 0.3671 **GTR+G** 7336.6772 32 0.3709

*virB6a** TVM+G 13509.0146 31 0.4682 TVM+G 13509.0146 31 0.4682

*virB6b** TVM+G 7278.5884 31 0.4399 TVM+G 7278.5884 31 0.4399

*virB6c** TVM+G 11665.4297 31 0.4453 TVM+G 11665.4297 31 0.4453

*virB6d* TVM+G 8891.6328 31 0.3971 **TVM+I+G** 8889.2998 32 4.6203

*virB6e* TVM+G 11767.5303 31 0.4677 **GTR+G** 11762.0645 32 0.4653

*virB8a** TVM+G 2408.8201 31 0.7862 TVM+G 2408.8201 31 0.7862

*virB8b** K81uf+G 2250.0146 29 0.5223 K81uf+G 2250.0146 29 0.5223

*virB9a* TrN+G 2178.2493 29 0.2671 **TIM+G** 2171.7344 30 0.2473

*virB9b* K81uf+G 1405.0817 29 0.6298 **TIM+G** 1402.4661 30 0.6523

*virB10** TIM+G 5343.832w5 30 0.4064 TIM+G 5343.8325 30 0.4064

*virB11* **TIM+G** 3017.8208 30 0.1581 TIM+I 3016.7952 30 0.6831

*virD4* **TIM+G**  5305.2666 30 0.2015 TIM+I+G 5303.5693 31 0.5987

*virB1** K81uf+G 3838.4644 29 0.4237 K81uf+G 3838.4644 29 0.4237

*virB2* HKY+G 1274.7262 28 0.4194 **TVM+G** 1270.8827 31 0.3582

*virB7* HKY+G 598.6234 28 0.6751 **TVM+I** 592.4268 31 0.4525

**^1^** Genes with asterisks had identical models and parameters predicted by hLRT and AIC.

**^2^** Bolded model depicts selected model for the ML analyses (when Hlrt and AIC did not agree).
